# Independent association of general and central adiposity with risk of gallstone disease: observational and genetic analyses

**DOI:** 10.3389/fendo.2024.1367229

**Published:** 2024-03-11

**Authors:** Min Zhang, Ye Bai, Yutong Wang, Huijie Cui, Wenqiang Zhang, Li Zhang, Peijing Yan, Mingshuang Tang, Yunjie Liu, Xia Jiang, Ben Zhang

**Affiliations:** ^1^ Clinical and Public Health Research Center, Chongqing Research Center for Prevention & Control of Maternal and Child Diseases and Public Health, Chongqing Health Center for Women and Children, Women and Children’s Hospital of Chongqing Medical University, Chongqing, China; ^2^ Gene Diagnosis Center, the First Affiliated Hospital of Jilin University, Jilin, China; ^3^ Department of Epidemiology and Health Statistics, Institute of Systems Epidemiology, and West China-PUMC C. C. Chen Institute of Health, West China School of Public Health and West China Fourth Hospital, Sichuan University, Chengdu, Sichuan, China

**Keywords:** gallstone disease, central obesity, observational association, genetic correlation, genome-wide cross-trait analysis

## Abstract

**Background:**

General obesity is a well-established risk factor for gallstone disease (GSD), but whether central obesity contributes additional independent risk remains controversial. We aimed to comprehensively clarify the effect of body fat distribution on GSD.

**Methods:**

We first investigated the observational association of central adiposity, characterized by waist-to-hip ratio (WHR), with GSD risk using data from UK Biobank (N=472,050). We then explored the genetic relationship using summary statistics from the largest genome-wide association study of GSD (*n_case_* =43,639, *n_control_* =506,798) as well as WHR, with and without adjusting for body mass index (BMI) (WHR: *n*=697,734; WHR_adj_BMI: *n*=694,649).

**Results:**

Observational analysis demonstrated an increased risk of GSD with one unit increase in WHR (HR=1.18, 95%CI=1.14-1.21). A positive WHR-GSD genetic correlation ( $r_{g}$ =0.41, *P*=1.42×10^-52^) was observed, driven by yet independent of BMI (WHR_adj_BMI: $r_{g}$ =0.19, *P*=6.89×10^-16^). Cross-trait meta-analysis identified four novel pleiotropic loci underlying WHR and GSD with biological mechanisms outside of BMI. Mendelian randomization confirmed a robust WHR-GSD causal relationship (OR=1.50, 95%CI=1.35-1.65) which attenuated yet remained significant after adjusting for BMI (OR=1.17, 95%CI=1.09-1.26). Furthermore, observational analysis confirmed a positive association between general obesity and GSD, corroborated by a shared genetic basis ( $r_{g}$ =0.40, *P*=2.16×10^-43^), multiple novel pleiotropic loci (N=11) and a causal relationship (OR=1.67, 95%CI=1.56-1.78).

**Conclusion:**

Both observational and genetic analyses consistently provide evidence on an association of central obesity with an increased risk of GSD, independent of general obesity. Our work highlights the need of considering both general and central obesity in the clinical management of GSD.

## Introduction

The role of general adiposity, as defined by body mass index (BMI), is well-characterized in the development of gallstone disease (GSD). Observational studies have identified an approximately 2-fold increased risk of GSD among individuals with general adiposity (1–3). Such a relationship has further been demonstrated by genetic studies using single nucleotide polymorphisms (SNP) as instrumental variables (IV). Three Mendelian randomization (MR) (4–6) have consistently quantified a 1.63-fold increased risk of GSD per-unit increment in BMI utilizing 97 BMI-associated IVs as well as 22,195 GSD cases and 472,022 non-cases.

Despite its well-established etiological role, general obesity alone is insufficient to explain the risk of obesity-related comorbidities, for which central or abdominal obesity should also be considered simultaneously for an improved clinical evaluation (7). Evidence, however, regarding central obesity, represented mainly by waist-to-hip ratio (WHR) and waist circumference (WC), and the development of GSD remains controversial. While some studies indicated that central obesity significantly increased GSD risk (effect sizes ranging from 1.10 to 21.36 for WHR, and from 1.01 to 3.94 for WC), others failed to support such findings (**Supporting Table S1**). Moreover, whether WHR or WC represents an independent risk factor in addition to BMI remains poorly understood. Among the only five observational studies that performed mutual adjustment, four reported a largely attenuated (26.5%-44.9%) yet significant effect of central obesity on GSD risk (8–11) while one study showed a null association (12). Furthermore, genetic studies largely supported a non-independent role of central obesity for which, despite MRs (4, 5) suggesting a positive effect of WHR (OR=1.007, 95%CI=1.001-1.013) and WC (OR=1.81, 95%CI=1.60-2.05) alone, the effect attenuated to null when taking BMI into consideration (WC_adj_BMI-GSD: OR=1.09, 95%CI=0.96-1.24). Nevertheless, these MRs used statistics from genome-wide association studies (GWAS) with a relatively small number of cases (*n*=22,195) or IVs (*n*=34-47) which may result in insufficient statistical power (5).

The independent role of central obesity thus the additional risk it brings, despite unclear in GSD, has been confirmed in other health-related conditions. For example, individuals characterized by high abdominal fat accumulation, even with normal BMI, showed a 1.31-fold increased risk of all-cause mortality (13) and an almost doubled risk of cardiometabolic factors (14), reflecting the importance of central obesity in predicting obesity-related disease risk.

Current progress in GWAS of GSD (with 43,639 cases and 506,798 controls) (15), WHR and BMI (with nearly 800,000 participants) (16) enables the utilization of a genome-wide cross-trait analysis, a well-established strategy that effectively characterizes the genetic architecture underlying observed phenotypic relationships, facilitating understandings to their biological mechanisms (17). In this study, we aimed to comprehensively investigate the role of obesity, both central and general, in the development of GSD through observational analysis and genome-wide cross-trait analysis.

## Materials and methods

**Figure 1:**
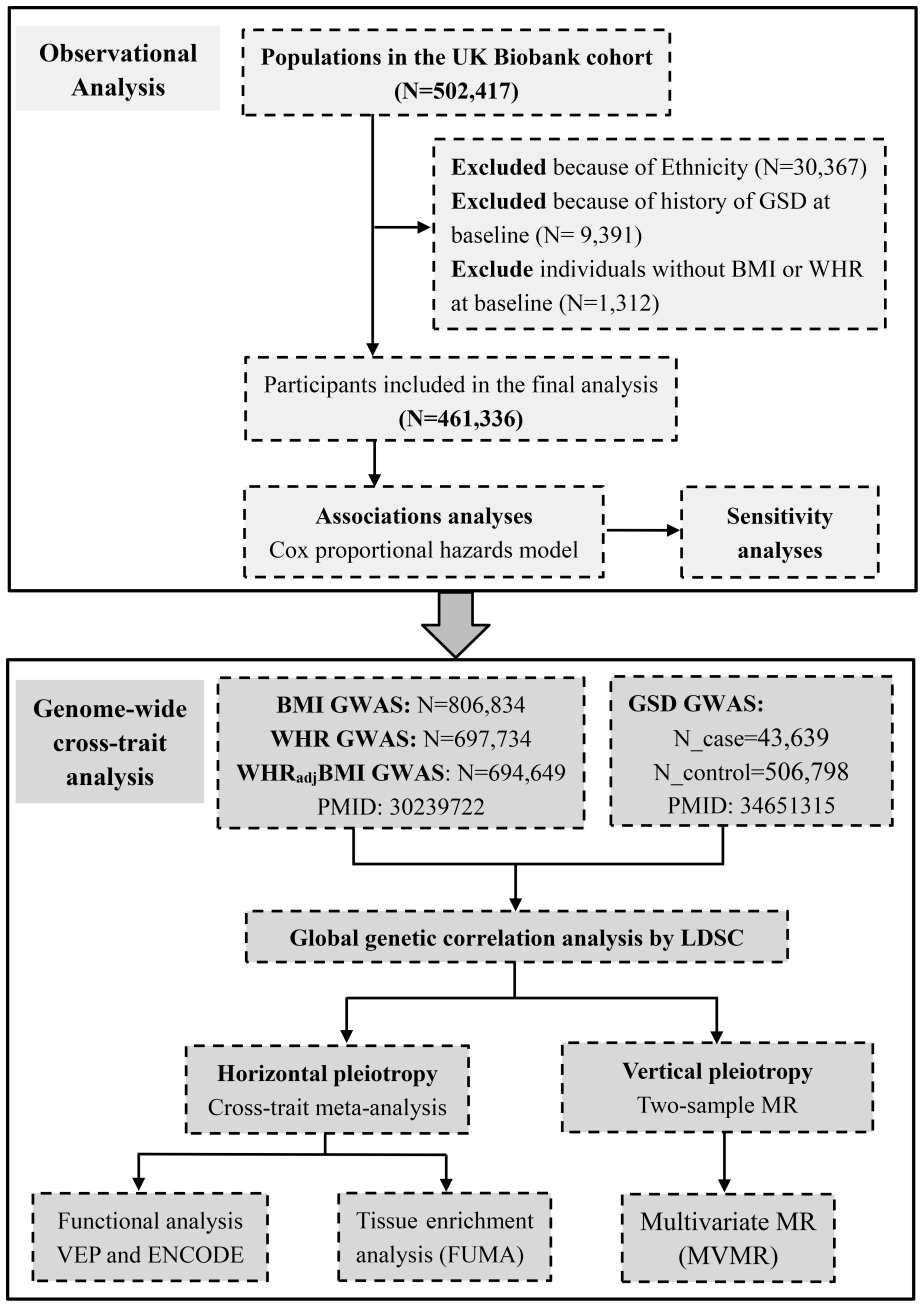
Conceptual framework of this study. GSD, gallstone disease; BMI, body mass index; WHR, waist-to-hip ratio; WHR_adj_BMI, waist-to-hip ratio adjusted for body mass index.

The conceptual framework of this study is shown in **Figure 1**. We utilized BMI to reflect general obesity. Despite both WHR and WC are common predictors for central obesity, WHR appeared to be more independent from BMI (Pearson’s *r*~0.4) than WC (Pearson’s *r*~0.8) (18) and had more IVs than WC (316 loci vs. 47 loci) (16, 19), and was thus used to represent central obesity. We further incorporated WHR_adj_BMI to represent the independent effect of central obesity after controlling for general obesity. We first explored the phenotypic relationship between adiposity and GSD using data from UK Biobank (UKB). We then conducted a genome-wide cross-trait analysis to clarify shared genetic basis, including a quantification of genetic correlation of BMI, WHR, WHR_adj_BMI with GSD, a cross-trait meta-analysis to identify pleiotropic loci, and a two-sample MR to infer putative causal associations.

### UK Biobank data

The UKB is a large prospective cohort study that recruits approximately half a million participants aged from 40 to 69 years at baseline from England, Wales and Scotland between 2006 and 2010. The National Health Service North West Multi-Centre Research Ethics Committee approved the study. The research complied with the Declaration of Helsinki. All participants provided written informed consent at recruitment, among which only 472,050 individuals of white descent were included. We defined a diagnosis of GSD based on the International Classification of Diseases, Tenth Revision (ICD-10) code “K80“ or Ninth Revision (ICD-9) code “574”. To avoid bias from population stratification, we excluded participants of Asian (or Asian British), Black (or Black British) and Mixed descent (n=30,367), and only considered the remaining 472,050 participants of white descent. We excluded 9,391 participants with a history of GSD at baseline as well as 1,312 individuals without BMI or WHR at baseline, leaving 461,336 eligible participants for analysis.

### Data sources of BMI, WHR, and WHR_adj_BMI

The hitherto largest GWAS for BMI, WHR, and WHR_adj_BMI was performed by meta-analyzing data from the Genetic Investigation of ANthropometric Traits (GIANT) consortium and UKB, which comprised up to 806,834 individuals of European ancestry (16). This GWAS meta-analyzed estimates across studies using a fixed-effect inverse-variance-weighted model and identified 670 BMI-associated index SNPs, 316 WHR-associated index SNPs, and 346 WHR_adj_BMI-associated index SNPs (*P<*5×10^−8^). We utilized these SNPs as IVs. We extracted the effect size and other relevant information of these IVs and downloaded the full set GWAS summary statistics. Details of data sources are listed in **Supporting Table S2**.

### Data sources of GSD

The hitherto largest GWAS for GSD was conducted by meta-analyzing data from UKB and FinnGen using a fixed-effect inverse-variance-weighted model (**Supporting Table S2**). This GWAS comprised 550,437 European participants, among which 43,639 individuals were diagnosed with GSD. The diagnostic codes included ICD-10, ICD-9, OPCS4, OPCS3, Read codes (primary care), and UKB self-reported codes (15). A total of 75 independent SNPs (*P<*5×10^−8^) were identified and used as IVs. We extracted the effect size and other relevant information of IVs and downloaded the full set GWAS summary statistics.

### Statistical analysis

#### Observational analysis

We presented baseline characteristics of study participants as mean ± standard deviation (SD) or median ± interquartile range (IQR) for continuous variables, and as frequency (percentage) for categorical variables.

We classified participants based on the World Health Organization BMI categories as underweight (<18.5 kg/m^2^), normal weight (18.5-25 kg/m^2^), overweight (25-30 kg/m^2^), obese class I (30-35 kg/m^2^), obese class II (35-40 kg/m^2^), and obese class III (>40 kg/m^2^) (20). We classified participants as central obesity when WHR was >0.90 in men and >0.85 in women (21). We also included BMI and WHR as continuous variables. We calculated person-year from baseline to GSD diagnosis, death, loss to follow-up, or end of follow-up, whichever occurred first. To test the phenotypic correlations of obesity with the risk of subsequent GSD, we constructed Cox proportional hazards regression model. We first adjusted for basic confounders including age, sex, assessment center, and the top 40 genetic principal components. We further adjusted for additional confounders including total cholesterol, triglycerides, high-density lipoprotein cholesterol (HDL-C), total bilirubin, diet, sedentary behavior, tea consumption, coffee consumption, current smoking, drinking, physical activity, income, Townsend deprivation index, type 2 diabetes, hypertension, liver disease, chronic kidney disease, Crohn’s disease, ulcerative colitis, cholecystitis, cholangitis, pancreatitis sleeve gastrectomy, use of anti-hyperlipidemia medication, anti-blood pressure medication, and insulin medication. Details of the confounders were present in **Supporting Methods**. We finally mutually adjusted for BMI and WHR. We further performed several sensitivity analyses to test the robustness of results. First, we excluded participants with less than a year of follow-up or a diagnosis of GSD within a year after enrollment (n=1,992). Then, we excluded participants who diagnosed with Crohn’s disease, ulcerative colitis, cholecystitis, cholangitis, and pancreatitis (n=4,948). In addition, we performed a subgroup analysis by sex.

We performed all statistical analyses using SAS software (version 9.4, SAS Institute, Cary, NC), and considered a two-sided *P<*0.05 as statistical significance.

#### Global genetic correlation analysis

Genome-wide genetic correlation ( $r_{g}$ ) quantifies the shared genetic basis between two traits that is independent of environmental confounders. We applied linkage disequilibrium score regression (LDSC) to estimate global genetic correlations of BMI, WHR, and WHR_adj_BMI with GSD using GWAS summary data. LDSC takes advantage of the fact that the GWAS effect size estimate for each locus represents the effects of all variants in linkage disequilibrium (LD) with that locus (22). The genetic correlation estimates range from –1 to +1, with –1 suggesting a complete negative correlation while +1 indicating a complete positive correlation. We adopted a Bonferroni-corrected *P*<0.017 (0.05/3) as statistical significance.

#### Cross-trait meta-analysis

We conducted a cross-trait meta-analysis by the method of cross-phenotype association (CPASSOC) to identify pleiotropy loci affecting both adiposity and GSD. CPASSOC integrates association evidence of multiple traits from multiple GWASs and therefore detects cross-phenotype associations. We adopted S_Het_ (rather than S_Hom_) which allows for heterogeneous effects of a trait from different studies (23). After CPASSOC, we utilized the PLINK clumping function to obtain independent shared loci, using parameters “–clump-p1 5e-8 –clump-p2 1e-5 –clump-r2 0.2 –clump-kb 500”. We considered a variant with *P*_single-trait_<1×10^-5^ (both traits) and *P*_CPASSOC_<5×10^-8^ as a significant pleiotropic variant.

We assigned each significant pleiotropic SNP into one of the four categories. First, a “known” SNP, defined as a shared SNP with *P*_single-trait_<5×10^-8^ for both single traits. Second, a “single-trait-driven” SNP, defined as a shared SNP with a *P*_single-trait_<5×10^-8^ for one of the two single traits. Third, an “LD-tagged” SNP, defined as a shared SNP in LD with index SNPs identified by single-trait GWAS(s) (LD *r* ^2^>0.2). Finally, a novel SNP, which was prioritized by us and of particular interest to us, was defined as a shared SNP neither driven by any single trait nor in LD with index SNPs identified by single-trait GWAS(s) (**Supporting Methods**). We additionally searched GWAS Catalog to test whether those shared SNPs were already reported as associated with phenotypes other than BMI, WHR, WHR_adj_BMI, or GSD.

To provide biological insights into the pleiotropic loci identified by CPASSOC, we mapped these SNPs to genes and conducted functional annotation by Ensembl Variant Effect Predictor (24) and Encyclopedia of DNA Elements (ENCODE) tool HaploReg v4.1 (25).

#### Tissue enrichment analysis

To identify tissues most relevant to shared genes, we conducted GTEx tissue enrichment analysis based on 54 tissue types available from GTEx (version 8) through functional mapping and annotation of genome-wide association studies (FUMA) GENE2FUNC process (26). We adopted Benjamin-Hochberg procedure to correct for multiple testing and considered a false discovery rate (FDR) corrected *P*<0.05 as statistical significance.

#### Mendelian randomization analysis

We finally conducted a two-sample MR to make causal inferences, following STROBE-MR guidelines (**Supporting Table S3**) (27), by using the relevant packages in R (version 3.6.3). MR uses genetic variants that are robustly associated with exposure as an instrument to assess causal relationships, and depends on three key assumptions (**Supporting Methods**). We calculated R^2^ to understand the proportion of variance in an “exposure” explained by IVs, and *F*-statistics to understand the strength of IVs. We also calculated statistical power based on an online web tool (https://sb452.shinyapps.io/power/). We applied an inverse-variance weighted (IVW) method as our primary MR approach. We further used MR-Egger regression and weighted median methods to test the robustness of results under relaxed model assumptions. We adopted a Bonferroni-corrected *P<*0.017 (0.05/3) as statistical significance. We determined an effect estimate as causal if it was statistically significant in IVW and remained directionally consistent in both MR-Egger regression and weighted median method.

We performed several sensitivity analyses to validify MR results. First, we excluded pleiotropic IVs which were associated with potential confounders according to NHGRI-EBI GWAS Catalog. Second, we excluded palindromic IVs, in which alleles are represented by the same pair of letters on the forward and the backward strands. Third, we performed a leave-one-out analysis where one variant was removed at a time and IVW was performed based on the remaining variants. Fourth, we applied MR-Pleiotropy Residual Sum and Outlier (MR-PRESSO) method to evaluate the presence of horizontal pleiotropy and to re-calculate causal effects after removing the detected outliers. Fifth, we conducted a reverse-directional MR evaluating the potential causal effect of GSD on obesity to rule out reverse causality. We also detected horizontal pleiotropy by MR-Egger intercept and considered significance when *P*<0.10. We further performed multivariate MR (MVMR), a statistical method that allows associations of SNPs with multiple phenotypes to be included in one model, permitting estimation of the direct impact of each phenotype on the outcome (28). Based on literature review, we included an overall healthy diet, childhood BMI, type 2 diabetes, HDL-C, low-density lipoprotein cholesterol, chronic kidney disease, non-alcoholic fatty liver disease, smoking, and alcohol drinking as potential confounders. Details of data sources of these potential confounders are listed in **Supporting Table S4**. To identify a potential sex difference, we performed a sex-specific MR using sex-specific IVs of BMI, WHR and WHR_adj_BMI.

## Results

### Observational analysis

The baseline characteristics of study participants are presented in **Supporting Table S5**. In total, participants were followed for 5,526,391 person-years with a mean follow-up of 11.98 ± 2.21 years, during which 15,283 individuals developed GSD. Consistent with previous findings, overweight defined by a BMI>25 kg/m^2^ was associated with an almost twofold increased risk of GSD (**Table 1**). We also observed an increasing trend of effect with the severity of obesity (P_trend_<0.0001). When treated as a continuous variable, each unit (five kg/m^2^) increase in BMI was also associated with an increased hazard of GSD (HR=1.31, 95%CI=1.28-1.34). The effect was slightly attenuated (8.58%) but remained significant (HR=1.28, 95%CI=1.25-1.31) after adjusting for WHR. Similar results were observed in both of the sensitivity analyses (**Supporting Table S6**). In the subgroup analysis, we observed a stronger effect size of overweight and general obesity on GSD in women than in men (*P* for interaction = 0.001) (**Supporting Table S7**).

**Table 1:** Observational relationships between obesity and the risk of subsequent GSD.

	Model 1		Model 2		Model 3	
	HR (95%CI)	*P*	HR (95%CI)	*P*	HR (95%CI)	*P*
General Obesity						
Normal (BMI: 18.5-25 kg/m^2^)	1.00 (ref)		1.00 (ref)		1.00 (ref)	
Underweight (BMI: >18.5 kg/m^2^)	0.83 (0.60, 1.16)	0.195	0.62 (0.37, 1.05)	0.076	0.63 (0.37, 1.07)	0.086
Overweight (BMI: 25-30 kg/m^2^)	1.92 (1.83, 2.01)	<0.0001	1.62 (1.53, 1.73)	<0.0001	1.55 (1.45, 1.65)	<0.0001
Obese class I (BMI: 30-35 kg/m^2^)	3.03 (2.88, 3.19)	<0.0001	2.21 (2.06, 2.37)	<0.0001	2.04 (1.90, 2.20)	<0.0001
Obese class II (BMI: 35-40 kg/m^2^)	4.06 (3.81, 4.33)	<0.0001	2.55 (2.33, 2.80)	<0.0001	2.34 (2.13, 2.57)	<0.0001
Obese class III (BMI: >40 kg/m^2^)	4.80 (4.40, 5.23)	<0.0001	2.56 (2.26, 2.91)	<0.0001	2.37 (2.09, 2.70)	<0.0001
*P* for trend	<0.0001		<0.0001		<0.0001	
BMI per 5 units	1.51 (1.49, 1.53)	<0.0001	1.31 (1.28, 1.34)	<0.0001	1.28 (1.25, 1.31)	<0.0001
Central obesity						
Normal (WHR: W<0.85, M<0.9)	1.00 (ref)		1.00 (ref)		1.00 (ref)	
Central obesity (WHR: W>0.85, M>0.9)	1.91 (1.85, 1.98)	<0.0001	1.48 (1.40, 1.55)	<0.0001	1.25 (1.18, 1.31)	<0.0001
WHR_per_SD	1.39 (1.38, 1.41)	<0.0001	1.26 (1.24, 1.29)	<0.0001	1.18 (1.14, 1.22)	<0.0001

Model 1: adjusted for age, sex, assessment center, and the top 40 genetic principal components.

Model 2: Model 1 with additional adjustment for total cholesterol, triglycerides, high-density lipoprotein cholesterol, total bilirubin, diet, sedentary behavior, tea consumption, coffee consumption, current smoking, drinking, physical activity, income, Townsend deprivation index, type 2 diabetes, hypertension, liver disease, chronic kidney disease, Crohn’s disease, ulcerative colitis, cholecystitis, cholangitis, pancreatitis sleeve gastrectomy, use of anti-hyperlipidemia medication, anti-blood pressure medication, and insulin medication.

Model 3: Model 2 with additional mutual adjustment for BMI and WHR.

W, women; M, men; BMI, body mass index; WHR, waist-to-hip ratio.

On the other hand, central obesity as defined by high WHR was associated with a significantly increased risk of GSD (HR=1.48, 95%CI=1.40-1.55) (**Table 1**). The effect remained significant but attenuated to some extent (43.1%) after adjusting for BMI (HR=1.25, 95%CI=1.18-1.31). A similar pattern was observed when replacing the binary variable with continuous variable (per-unit change WHR-GSD: HR=1.27, 95%CI=1.25-1.30; after adjusting for BMI: HR=1.18, 95%CI=1.14-1.21). Similar results were observed in both of the sensitivity analyses (**Supporting Table S6**). We observed no significant sex difference in the effect of central obesity on GSD (**Supporting Table S7**).

### Global genetic correlation

As shown in **Table 2**, for both BMI ( $r_{g}$ =0.40, *P=*2.16×10^-43^) and WHR ( $r_{g}$ =0.41, *P=*1.42×10^-52^), we observed a comparably strong genetic correlation with GSD. The WHR-GSD estimate, however, attenuated to half (53.7%) when the effect of BMI was removed (WHR_adj_BMI-GSD: $r_{g}$ =0.19, *P=*6.89×10^-16^), yet remained statistically significant. All estimates withstood Bonferroni correction.

**Table 2:** Genome-wide genetic correlation between GSD and obesity-related traits.

Trait 1	Trait 2	*r*_g_	*r*_g__se	*r*_g__*P*	gcov	gcov_se
GSD	BMI	0.40	0.029	2.16×10^-43^	0.100	0.008
GSD	WHR	0.41	0.027	1.42×10^-52^	0.080	0.007
GSD	WHR_adj_BMI	0.19	0.024	6.89×10^-16^	0.031	0.007

$r_{g}$ , genetic correlation; se, standard error; gcov, genetic covariance; GSD, gallstone disease; BMI, body mass index; WHR, waist-to-hip ratio; WHR_adj_BMI, waist-to-hip ratio adjusted for body mass index.

### Cross-trait meta-analysis

For BMI and GSD, we identified a total of 44 pleiotropic loci (**Table 3**, **Supporting Table S8**). After excluding “known”, “single-trait-driven”, or “LD-tagged” loci, we identified 11 novel loci. Notably, the most significant pleiotropic locus was rs11672660 (*P_CPASSOC_=*8.10×10^-72^) mapped to *GIPR*, a gene associated with glucose tolerance (29). The most significant novel pleiotropic locus was rs12900395 (*P_CPASSOC_* =8.26×10^-12^) mapped to *PSTPIP1*, a gene involved in immunoregulatory functions (30).

**Table 3:** Cross-trait meta-analysis identified novel pleiotropic loci between obesity-related traits and GSD.

SNP	CHR : BP	A1/A2	Obesity		GSD		*P*_CPASSOC_	Linear closest genes	GENCODE genes
			BETA	*P*	BETA	*P*			
BMI and GSD									
rs10424365	19:19310527	G/A	0.012	5.87×10^-07^	0.059	1.08×10^-06^	2.26×10^-11^	RFXANK	RFXANK
rs11065363	12:121388498	T/C	0.011	6.32×10^-06^	0.049	3.59×10^-06^	9.83×10^-10^	–	18kb 3’ of HNF1A-AS1
rs12900395	15:77310345	G/C	0.009	9.08×10^-08^	0.035	5.92×10^-06^	8.26×10^-12^	PSTPIP1	PSTPIP1
rs1374915	3:71668037	C/T	-0.010	3.58×10^-07^	-0.038	1.03×10^-06^	1.19×10^-11^	–	35kb 5’ of FOXP1
rs147233090	15:44028047	T/C	-0.029	5.49×10^-06^	0.142	1.33×10^-07^	9.50×10^-11^	CATSPER2P1	1.6kb 5’ of U6
rs3744405	17:7193255	A/G	0.008	4.11×10^-06^	0.035	3.47×10^-06^	5.62×10^-10^	YBX2	YBX2
rs4886838	15:77158170	T/C	0.010	6.14×10^-06^	0.046	2.30×10^-06^	7.02×10^-10^	SCAPER	SCAPER
rs6544597	2:43013593	G/T	0.009	3.54×10^-06^	0.045	2.22×10^-07^	7.46×10^-11^	HAAO	HAAO
rs76637437	10:65079361	C/T	-0.015	9.35×10^-07^	0.062	1.10×10^-06^	4.07×10^-11^	JMJD1C	JMJD1C
rs9571577	13:66937503	G/A	0.009	2.70×10^-06^	0.036	1.69×10^-06^	2.04×10^-10^	PCDH9	PCDH9
rs9625962	22:44326272	C/T	-0.013	1.80×10^-06^	-0.050	9.29×10^-07^	8.17×10^-11^	PNPLA3	PNPLA3
WHR and GSD									
rs228757	17:42164885	C/G	-0.010	4.24×10^-07^	0.044	6.45×10^-07^	4.72×10^-12^	HDAC5	HDAC5
rs2457445	10:64781226	A/G	-0.010	1.74×10^-07^	0.043	8.38×10^-08^	3.21×10^-13^	–	89kb 5’ of U6
rs2686189	8:11655229	T/C	-0.009	8.61×10^-06^	-0.038	3.36×10^-06^	6.69×10^-10^	FDFT1	FDFT1
rs6889220	5:176693888	A/G	0.014	4.28×10^-07^	0.051	6.79×10^-06^	3.25×10^-11^	NSD1	NSD1
WHR_adj_BMI and GSD									
rs55666908	10:65273534	A/G	0.014	3.85×10^-06^	-0.056	4.71×10^-06^	3.03×10^-10^	–	7.6kb 5’ of REEP3

A1/A2, effect allele/other allele; BMI, body mass index; GSD, gallstone disease; WHR, waist-to-hip ratio; WHR_adj_BMI, waist-to-hip ratio adjusted for body mass index. Linear closest genes of index SNPs were mapped by using VEP; GENCODE genes of index SNPs were mapped by HeploReg V4.1.

For WHR and GSD, we identified a total of 24 pleiotropic loci (**Table 3**, **Supporting Table S9**), among which four were novel. Notably, the most significant pleiotropic SNP was rs1558902 (*P_CPASSOC_* =9.68×10^-128^) mapped to *FTO*, a gene known to affect obesity-related phenotypes (31). The most significant novel pleiotropic SNP was rs2457445 (*P_CPASSOC_=*3.21×10^-13^), located in an intergenic region. Among the 24 shared loci, seven were also found to be shared by BMI-GSD, ten were in LD (*r* ^2^>0.2) with BMI-GSD shared loci, and the remaining seven were unique. For WHR_adj_BMI and GSD, we identified 24 pleiotropic SNPs when removing the effect of BMI (**Table 3**, **Supporting Table S9**), among which one locus was novel. The most significant pleiotropic SNP was rs55747707 (*P*_CPASSOC_=5.05×10^-34^) mapped to *MLXIPL* (also known as *CHREBP*), a gene involved in HDL-C and triglyceride synthesis (32). The novel pleiotropic locus, rs62130338 (*P*_CPASSOC_=3.03×10^-10^), located in *JMJD1C* gene. Among the 24 shared loci, four were also found to be shared by BMI-GSD, eight were in LD (*r* ^2^>0.2) with BMI-GSD shared loci, three were also found to be shared by WHR-GSD, and the remaining nine were unique.

We further searched GWAS Catalog to test whether those shared SNPs were already reported to associate with other phenotypes. Among the 16 novel shared SNP, most of the loci were completely novel SNPs, two (rs147233090 for BMI-GSD, rs9625962 for WHR-GSD) of these were also associated with triacylglycerol, hemoglobin levels, or calcium levels (**Supporting Table S10**). Other detailed annotations of each individual pleiotropic SNP are shown in **Supporting Table S11**.

### Tissue enrichment analysis

As shown in **Supporting Figure S1**, we observed significant enrichment in liver and heart atrial appendage tissues for the expression of genes shared by BMI and GSD. For WHR and GSD shared genes, we identified significant enrichment in brain caudate basal ganglia, brain putamen basal ganglia, and heart left ventricle tissues, however, we observed no significant tissue enrichment after adjusting for BMI (WHR_adj_BMI-GSD).

### The causal relationship between BMI, WHR, WHR_adj_BMI and GSD

The calculated R^2^ and *F*-statistic suggested robust IVs (**Supporting Table S2**). With the current sample size for GSD (*n_total_* =550,437, *n_case_* =43,639) and calculated R^2^, our MR had more than 80% statistical power to detect an estimate of 1.20 on GSD for 1-unit increase in BMI and WHR.

**Figure 2:**
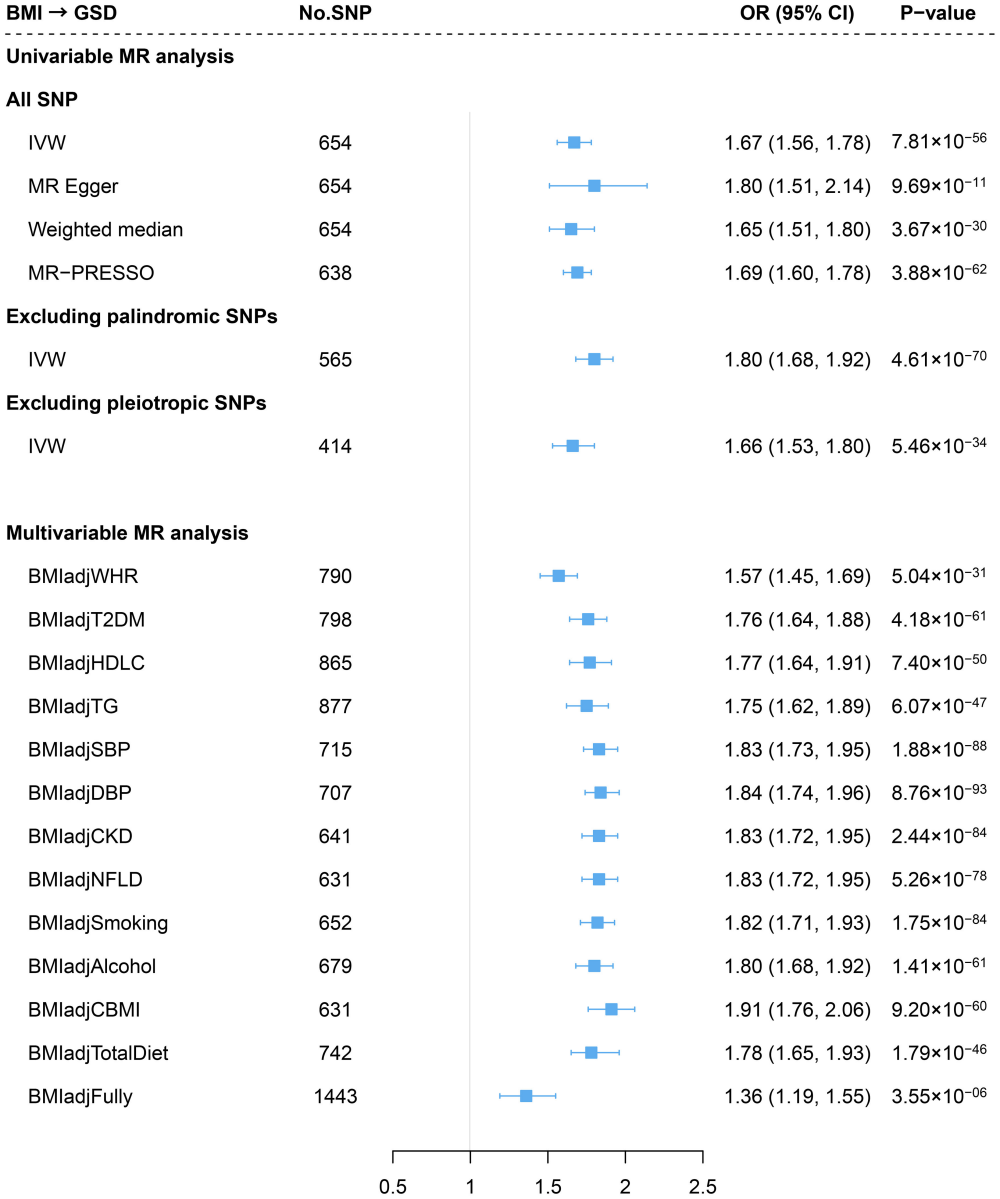
Estimates of causal relationships between general obesity and gallstone disease risk. GSD, gallstone disease; BMI, body mass index; WHR, waist-to-hip ratio; T2DM, type 2 diabetes mellitus; HDLC, high-density lipoprotein cholesterol; TG, triglyceride; SBP, systolic blood pressure; DBP, diastolic blood pressure; CKD, chronic kidney disease; NFLD, non-alcoholic fatty liver disease; CBMI, childhood body mass index.

Consistent with previous findings, genetically predicted BMI was associated with an increased risk of GSD (OR=1.67, 95%CI=1.56-1.78) using an updated number of IVs (**Figure 2**). The effect did not alter in weighted median or MR-Egger method. We observed no sign of horizontal pleiotropy (*P* for MR-Egger intercept=0.37). Removing palindromic variants, pleiotropic variants or outliers (MR-PRESSO) yielded to a similar result (**Figure 2**) and the leave-one-out analysis indicated no outlying SNP (**Supporting Figure S2**). The effect size attenuated slightly after adjusting for WHR (BMI_adj_WHR: OR=1.57, 95%CI=1.45-1.69; risk reduction: 9.5%) (**Figure 2**). Further adjustment of other confounders did not substantially alter the results (fully adjusted: OR=1.36, 95%CI=1.19-1.55) (**Figure 2**). No significant difference was observed in the risk of GSD using sex-specific IVs of BMI (women: OR=1.66; men: OR=1.64, *P* for interaction=0.939) (**Supporting Figure S3**).

**Figure 3:**
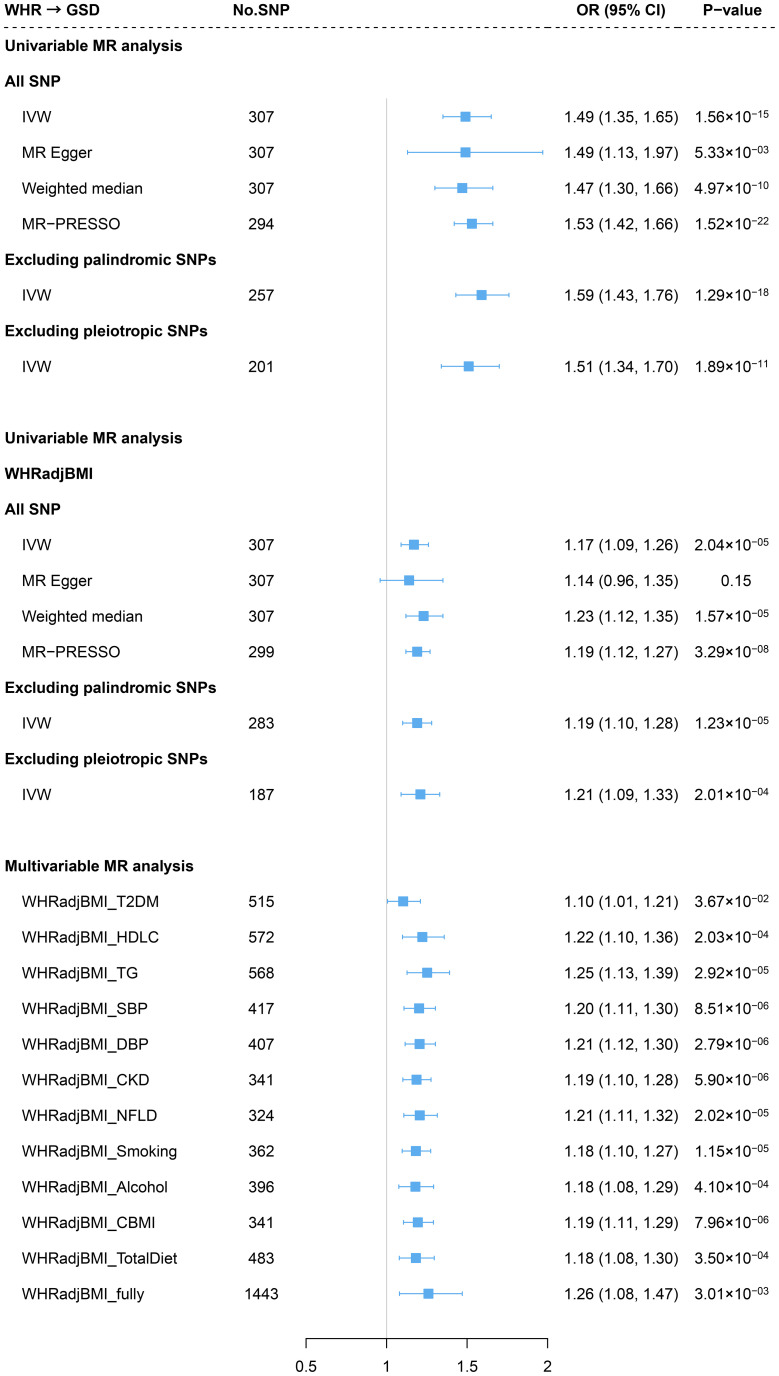
Estimates of causal relationships between central obesity and gallstone disease risk. GSD, gallstone disease; WHR, waist-to-hip ratio; BMI, body mass index; WHRadjBMI, waist-to-hip ratio adjusted for body mass index; T2DM, type 2 diabetes mellitus; HDLC, high-density lipoprotein cholesterol; TG, triglyceride; SBP, systolic blood pressure; DBP, diastolic blood pressure; CKD, chronic kidney disease; NFLD, non-alcoholic fatty liver disease; CBMI, childhood body mass index.

Genetically predicted WHR was associated with a significantly increased risk of GSD (OR=1.49, 95%CI=1.35-1.65) using 307 IVs. The association remained consistent across the weighted median method, MR-Egger approach, and sensitivity analyses removing palindromic IVs, pleiotropic IVs, or outliers (MR-PRESSO) (**Figure 3**). We did not identify any sign of horizontal pleiotropy (*P* for MR-Egger intercept=0.99). The leave-one-out analysis also showed no outlying SNP (**Supporting Figure S2**). When adjusting for BMI, the effect decreased by 28.1% yet remained statistically significant (WHR_adj_BMI: OR=1.17, 95%CI=1.09-1.26) (**Figure 3**). When additional adjusted for other confounders, the effect of WHR independent of BMI also remain consistent (fully adjusted: OR=1.26, 95%CI=1.08-1.47) (**Figure 3**). Despite the causal estimate of genetically determined WHR with GSD was stronger in men (OR=1.89) than in women (OR=1.31), this difference became less pronounced after adjusting for BMI (women: OR=1.14; men: OR=1.22, *P* for interaction=0.339). (**Supporting Figure S3**).

On the contrary, we did not observe any significant association of genetically predicted GSD with the risk of BMI, WHR, or WHR_adj_BMI in the reverse-direction MR (**Supporting Figure S4**).

## Discussion

As far as we understand, this is the most comprehensive observational and genetic analysis investigating the phenotypic association and genetic correlation between different types of obesity and GSD. Consistent with previous findings, we confirmed a major etiological role of general obesity in the development of GSD. We further observed an independent effect of central obesity on GSD, corroborated by the significant phenotypic association as well as the shared genetic basis after controlling for the effect of BMI.

Our findings on general obesity and GSD are consistent with those from existing studies yet greatly expand previous results. Firstly, through utilizing the hitherto largest genetic data as well as an advanced analysis strategy, our study revealed a shared genetic basis underlying general obesity and GSD. Furthermore, the 44 pleiotropic loci for BMI and GSD indicated a shared biological mechanism underlying general obesity and GSD. In addition, as to the assessment of causal relationship, by using an almost doubled GSD cases (43,639 vs. 22,195) and significantly augmented number of IVs of BMI (>565 vs. 97) as compared to previous MRs (4–6), the statistical power in our MR was greatly improved. Importantly, after adjusting for WHR and other important confounders (such as diet, smoking, alcohol, CKD, and NFLD) which have not been done in previous MRs, the robust causal estimates also validated the reliability of the effect of general obesity on GSD.

In addition to general obesity, central obesity has also been linked closely with various health conditions. For example, a cohort study showed men with normal BMI but with central obesity had twice the mortality risk compared to those who were overweight (HR=2.24) or obesity (HR=2.42) defined only by BMI (33). Despite its independent role identified for multiple diseases, central obesity in the development of GSD remains controversial. For example, Tsai et al. (9) observed a positive association between WHR and GSD, while Boland et al. (11) reported a null association in men; furthermore Kodama et al. (12) found that the positive association attenuated to null after controlling for BMI. These contradictory findings, however, can be largely ascribed to a small number of GSD cases (ranging from 41 to 8,477). Our results, based on the hitherto largest sample size in terms of both cases and non-GSD referents (N_total_=461,336, N_GSD_=15,283), enable an accurate quantification on the effect of WHR with a greatly enhanced statistical power. The accessibility to a large number of covariates also allows the investigation of an independent effect of WHR through adjusting for multiple confounders (e.g., genetic principal components and medications usage), which previous studies did not have a chance for. Findings from observational analysis has further been validated by genetic associations. Indeed, a previous MR quantified the causal association between WHR and GSD and found a modest effect of 1.007 which might be of limited clinical relevance (5). That study, however, was hampered by a small number of IVs (*n*=34) and inadequate GSD cases (*n*=14,723). With a ten-times increased number of IVs as well as a greatly enlarged number of cases, we were able to quantify the effect of WHR with a decent statistical power. We were also able to reveal the independent role of WHR through controlling for important confounders including BMI and others. Our analysis demonstrates an independent effect of WHR alone on the pathological pathways leading to GSD while largely ruling out reverse causality.

Positive genetic correlation identified by LDSC suggests shared genetic basis underlying central obesity and GSD, which can be decomposed into horizontal and vertical pleiotropy. In addition to the causal relationship (vertical pleiotropy), results from cross-trait meta-analysis indicate biological pleiotropy (horizontal pleiotropy). We highlight several shared loci of interest, which showing mechanistic pathways outside of general obesity. First of all, SNP rs228757 was a missense located in *HDAC5*, and this gene was specifically shared by WHR and GSD, encoding a histone deacetylase that plays an important role in the development of brain, heart, and muscle (34). Lenoir et al. found an increase in insulin-producing β-cell mass in the pancreas of *HDAC5*-knockout mice while a decrease in *HDAC5*-overexpressed mice, demonstrating a suppressing role of *HDAC5* in insulin production (35). Such an effect on insulin action was also observed in muscle cells (36, 37). Studies have shown that insulin resistance is associated with both GSD and central obesity. For example, Chang et al. (38) observed a significantly increased risk of gallstones with insulin resistance in non-diabetic men, regardless of general obesity, suggesting its independent role in GSD. When conducting genetic pathway analysis utilizing WHR_adj_BMI-associated loci, Shungin et al. (19) implicated insulin resistance as a process affecting body fat distribution. Furthermore, Kullmann et al. (39) observed a positive association of insulin responsiveness of the hypothalamus with visceral but not subcutaneous fat, suggesting brain insulin resistance appeared to be a determinant of abdominal obesity. Taken together, insulin resistance might play an important role in pathophysiological mechanisms of GSD caused by central obesity.

For SNPs shared by WHR_adj_BMI and GSD, rs55666908 was a novel pleiotropic SNP that maps to *JMJD1C*. *JMJD1C* is a candidate histone demethylase and is thought to be a coactivator for key transcription factors, and its involvement in GSD and central obesity has rarely been studied. Lipid synthesis-associated protein FABP5 was identified as a specific interacting protein of JMJD1C and binds to the jumonji domain of JMJD1C, suggesting its potential role in GSD and central obesity (JMJD1C-regulated lipid synthesis) (40). Another pleiotropic SNP, rs601338, maps to *FUT2*. *FUT2* was specifically shared by WHR_adj_BMI and GSD, but its involvement in GSD and central obesity has rarely been studied. By using large-scale genetic data, common variants of *FUT2* have been identified to be associated with primary sclerosing cholangitis (41) and concentrations of liver enzymes (42), which might be potential indicators for gallstones. Using *FUT2*-knockout mice, Maroni et al. (43) found elevated levels of systemic bile salt, further suggesting its potential lithogenic role. Furthermore, the GG carriers of rs601338 in *FUT2* had an increased number of gut bacteria (such as *Escherichia*) that involved in the short-chain fatty acids synthesis compared to carriers of other variants (44), which has shown to be related to visceral fat accumulation (45), suggesting the potential role of *FUT2* in central obesity. More researches are needed to determine the precise mechanisms underlying central obesity and GSD.

Our findings provide important clinical and public health implications. First of all, our study further verified that obesity and GSD are inherently linked through biological pleiotropy and common origin. Integrating care targeting both trait, including continuous health promotion, disease prevention, screening, and management, should thus be provided for human to reduce the burden brought by both disease. Then, almost all current public health guidelines focus specifically on maintaining a normal weight while rarely address body fat distribution (46). Our findings emphasize the demand for future public health guidelines to take central obesity into consideration. In addition, our findings demonstrate that combining general and central obesity may better stratify high-risk group of GSD than using either measurement alone in the clinical practice.

Our study has some strength. First, the effect of body fat distribution on GSD was elucidated comprehensively through observational analysis and genetic analysis. Second, a genetic association between central obesity and gallstone disease was observed, independent of general obesity. Third, through the cross-trait meta-analysis, four new common loci had been identified, revealing the biological mechanism between central obesity and gallstone disease. There are several limitations. First, as GSD is known to be more prevalent in women than in men, we tried to identify a potential sex difference through a sex-specific MR. No consistent sex difference was found in the effect of both central and general obesity on GSD. Nonetheless, the GWAS of GSD was derived from a sex-combined population, which might bias the MR estimates. Detailed sex-specific genetic analysis is needed to clarify a potential sex-preponderance. Second, observational study using data from UKB has the potential for retrospective and information bias. Third, the GWASs used in the genetic analysis came from different studies, and thus there may be heterogeneity and confounding effects. Fourth, our findings were restricted to European population to control for population stratification, this might also limit the generalizability to other populations. Fifth, although our study identified shared genes and tissues with different types of obesity and GSD, they relied on functional datasets and algorithms. Further experimental researches are needed to illustrate the pathophysiological mechanisms.

## Conclusions

To conclude, using data from a large-scale prospective cohort as well as summary statistics from the largest GWASs, our study demonstrates an independent role of central obesity in the development of GSD, in addition to confirming the effect of general obesity. Using advanced statistical genetics approaches, our study provides novel insights into the etiological basis of GSD with the involvement of different types of obesity. These findings improve the prevention of GSD by emphasizing the need for body-fat distribution management in addition to weight management.

## Data availability statement

Publicly available datasets were analyzed in this study. This data can be found here: www.biobank.ac.uk and https://zenodo.org/record/1251813#.X37_UZMzbLs.

## Ethics statement

The studies involving humans were approved by UK Biobank cohort study had obtained ethics approval from the North West Multi-Centre Research Ethics Committee which covers the UK (approval number: 11/NW/0382) and had obtained informed consent from all participants. The current study was approved by the UK Biobank access management board (approval number: 50538). The GWAS summary statistics used in the present study are aggregated level of data which do not contain any personal identifiers. The original GWAS have obtained ethical approval from relevant ethics review committees. The studies were conducted in accordance with the local legislation and institutional requirements. The participants provided their written informed consent to participate in this study.

## Author contributions

MZ: Writing – original draft, Visualization, Validation, Methodology, Investigation, Data curation. YB: Writing – original draft, Visualization, Methodology, Data curation. YW: Writing – review & editing, Methodology, Investigation. HC: Writing – review & editing, Visualization, Investigation. WZ: Writing – review & editing, Visualization, Methodology, Data curation. LZ: Writing – review & editing, Visualization, Validation. PY: Writing – review & editing, Validation, Methodology. MT: Writing – review & editing, Software, Methodology. YL: Writing – review & editing, Project administration, Investigation. XJ: Writing – review & editing, Validation, Supervision, Resources, Project administration, Funding acquisition, Conceptualization. BZ: Writing – review & editing, Supervision, Resources, Funding acquisition, Formal Analysis, Conceptualization.

## References

[1] Stampfer MJ Maclure KM Colditz GA Manson JE Willett WC. Risk of symptomatic gallstones in women with severe obesity. Am J Clin Nutr. (1992) 55:652–8. doi: 10.1093/ajcn/55.3.652 1550039

[2] Banim PJ Luben RN Bulluck H Sharp SJ Wareham NJ Khaw KT. The aetiology of symptomatic gallstones quantification of the effects of obesity, alcohol and serum lipids on risk. Epidemiological and biomarker data from a UK prospective cohort study (EPIC-Norfolk). Eur J Gastroenterol Hepatol. (2011) 23:733–40. doi: 10.1097/MEG.0b013e3283477cc9 21623190

[3] Katsika D Tuvblad C Einarsson C Lichtenstein P Marschall HU. Body mass index, alcohol, tobacco and symptomatic gallstone disease: a Swedish twin study. J Intern Med. (2007) 262:581–7. doi: 10.1111/j.1365-2796.2007.01860.x 17908165

[4] Yuan S Gill D Giovannucci EL Larsson SC. Obesity, type 2 diabetes, lifestyle factors, and risk of gallstone disease: A Mendelian randomization investigation. Clin Gastroenterol Hepatol. (2022) 20:e529–37. doi: 10.1016/j.cgh.2020.12.034 33418132

[5] Chen L Yang H Li H He C Yang L Lv G. Insights into modifiable risk factors of cholelithiasis: A Mendelian randomization study. Hepatology. (2022) 75:785–96. doi: 10.1002/hep.32183 PMC9300195 34624136

[6] Stender S Nordestgaard BG Tybjaerg-Hansen A. Elevated body mass index as a causal risk factor for symptomatic gallstone disease: a Mendelian randomization study. Hepatology. (2013) 58:2133–41. doi: 10.1002/hep.26563 23775818

[7] Bosomworth NJ. Normal-weight central obesity: Unique hazard of the toxic waist. Can Fam Physician. (2019) 65:399–408. 31189627 PMC6738397

[8] Hou L Shu XO Gao YT Ji BT Weiss JM Yang G. Anthropometric measurements, physical activity, and the risk of symptomatic gallstone disease in Chinese women. Ann Epidemiol. (2009) 19:344–50. doi: 10.1016/j.annepidem.2008.12.002 PMC3013626 19362277

[9] Tsai CJ Leitzmann MF Willett WC Giovannucci EL. Central adiposity, regional fat distribution, and the risk of cholecystectomy in women. Gut. (2006) 55:708–14. doi: 10.1136/gut.2005.076133 PMC1856127 16478796

[10] Tsai CJ Leitzmann MF Willett WC Giovannucci EL. Prospective study of abdominal adiposity and gallstone disease in US men. Am J Clin Nutr. (2004) 80:38–44. doi: 10.1093/ajcn/80.1.38 15213025

[11] Boland LL Folsom AR Rosamond WD Atherosclerosis Risk in Communities Study I. Hyperinsulinemia, dyslipidemia, and obesity as risk factors for hospitalized gallbladder disease. A prospective study. Ann Epidemiol. (2002) 12:131–40. doi: 10.1016/s1047-2797(01)00260-5 11880221

[12] Kodama H Kono S Todoroki I Honjo S Sakurai Y Wakabayashi K. Gallstone disease risk in relation to body mass index and waist-to-hip ratio in Japanese men. Int J Obes Relat Metab Disord. (1999) 23:211–6. doi: 10.1038/sj.ijo.0800781 10078858

[13] Mohammadian Khonsari N Khashayar P Shahrestanaki E Kelishadi R Mohammadpoor Nami S Heidari-Beni M. Normal weight obesity and cardiometabolic risk factors: A systematic review and meta-analysis. Front Endocrinol (Lausanne). (2022) 13:857930. doi: 10.3389/fendo.2022.857930 35399938 PMC8987277

[14] Sun Y Liu B Snetselaar LG Wallace RB Caan BJ Rohan TE. Association of normal-weight central obesity with all-cause and cause-specific mortality among postmenopausal women. JAMA Netw Open. (2019) 2:e197337. doi: 10.1001/jamanetworkopen.2019.7337 31339542 PMC6659146

[15] Fairfield CJ Drake TM Pius R Bretherick AD Campbell A Clark DW. Genome-wide analysis identifies gallstone-susceptibility loci including genes regulating gastrointestinal motility. Hepatology. (2022) 75:1081–94. doi: 10.1002/hep.32199 34651315

[16] Pulit SL Stoneman C Morris AP Wood AR Glastonbury CA Tyrrell J. Meta-analysis of genome-wide association studies for body fat distribution in 694 649 individuals of European ancestry. Hum Mol Genet. (2019) 28:166–74. doi: 10.1093/hmg/ddy327 PMC6298238 30239722

[17] Zhu Z Hasegawa K Camargo CA Jr. Liang L. Investigating asthma heterogeneity through shared and distinct genetics: Insights from genome-wide cross-trait analysis. J Allergy Clin Immunol. (2021) 147:796–807. doi: 10.1016/j.jaci.2020.07.004 32693092 PMC7368660

[18] Wei M Gaskill SP Haffner SM Stern MP. Waist circumference as the best predictor of noninsulin dependent diabetes mellitus (NIDDM) compared to body mass index, waist/hip ratio and other anthropometric measurements in Mexican Americans–a 7-year prospective study. Obes Res. (1997) 5:16–23. doi: 10.1002/j.1550-8528.1997.tb00278.x 9061711

[19] Shungin D Winkler TW Croteau-Chonka DC Ferreira T Locke AE Magi R. New genetic loci link adipose and insulin biology to body fat distribution. Nature. (2015) 518:187–96. doi: 10.1038/nature14132 PMC4338562 25673412

[20] Pai MP Wilcox MH Chitra S McGovern PC. Safety and efficacy of omadacycline by BMI categories and diabetes history in two Phase III randomized studies of patients with acute bacterial skin and skin structure infections. J Antimicrob Chemother. (2021) 76:1315–22. doi: 10.1093/jac/dkaa558 PMC8050767 33458763

[21] Cornier MA Dabelea D Hernandez TL Lindstrom RC Steig AJ Stob NR. The metabolic syndrome. Endocr Rev. (2008) 29:777–822. doi: 10.1210/er.2008-0024 18971485 PMC5393149

[22] Bulik-Sullivan BK Loh PR Finucane HK Ripke S Yang J Schizophrenia Working Group of the Psychiatric Genomics C. LD Score regression distinguishes confounding from polygenicity in genome-wide association studies. Nat Genet. (2015) 47:291–5. doi: 10.1038/ng.3211 PMC4495769 25642630

[23] Zhu X Feng T Tayo BO Liang J Young JH Franceschini N. Meta-analysis of correlated traits via summary statistics from GWASs with an application in hypertension. Am J Hum Genet. (2015) 96:21–36. doi: 10.1016/j.ajhg.2014.11.011 25500260 PMC4289691

[24] McLaren W Gil L Hunt SE Riat HS Ritchie GR Thormann A. The ensembl variant effect predictor. Genome Biol. (2016) 17:122. doi: 10.1186/s13059-016-0974-4 27268795 PMC4893825

[25] Ward LD Kellis M. HaploReg: a resource for exploring chromatin states, conservation, and regulatory motif alterations within sets of genetically linked variants. Nucleic Acids Res. (2012) 40:D930–4. doi: 10.1093/nar/gkr917 PMC3245002 22064851

[26] Watanabe K Taskesen E van Bochoven A Posthuma D. Functional mapping and annotation of genetic associations with FUMA. Nat Commun. (2017) 8:1826. doi: 10.1038/s41467-017-01261-5 29184056 PMC5705698

[27] Skrivankova VW Richmond RC Woolf BAR Yarmolinsky J Davies NM Swanson SA. Strengthening the reporting of observational studies in epidemiology using Mendelian randomization: the STROBE-MR statement. JAMA. (2021) 326:1614–21. doi: 10.1001/jama.2021.18236 34698778

[28] Sanderson E Davey Smith G Windmeijer F Bowden J. An examination of multivariable Mendelian randomization in the single-sample and two-sample summary data settings. Int J Epidemiol. (2019) 48:713–27. doi: 10.1093/ije/dyy262 PMC6734942 30535378

[29] Saxena R Hivert MF Langenberg C Tanaka T Pankow JS Vollenweider P. Genetic variation in GIPR influences the glucose and insulin responses to an oral glucose challenge. Nat Genet. (2010) 42:142–8. doi: 10.1038/ng.521 PMC2922003 20081857

[30] Boursier G Piram M Rittore C Sarrabay G Touitou I. Phenotypic associations of PSTPIP1 sequence variants in PSTPIP1-associated autoinflammatory diseases. J Invest Dermatol. (2021) 141:1141–7. doi: 10.1016/j.jid.2020.08.028 33218716

[31] Scuteri A Sanna S Chen WM Uda M Albai G Strait J. Genome-wide association scan shows genetic variants in the FTO gene are associated with obesity-related traits. PloS Genet. (2007) 3:e115. doi: 10.1371/journal.pgen.0030115 17658951 PMC1934391

[32] Haslam DE Peloso GM Guirette M Imamura F Bartz TM Pitsillides AN. Sugar-sweetened beverage consumption may modify associations between genetic variants in the CHREBP (Carbohydrate responsive element binding protein) locus and HDL-C (High-density lipoprotein cholesterol) and triglyceride concentrations. Circ Genom Precis Med. (2021) 14:e003288. doi: 10.1161/CIRCGEN.120.003288 34270325 PMC8373451

[33] Sahakyan KR Somers VK Rodriguez-Escudero JP Hodge DO Carter RE Sochor O. Normal-weight central obesity: implications for total and cardiovascular mortality. Ann Intern Med. (2015) 163:827–35. doi: 10.7326/M14-2525 PMC4995595 26551006

[34] Mathias RA Guise AJ Cristea IM. Post-translational modifications regulate class IIa histone deacetylase (HDAC) function in health and disease. Mol Cell Proteomics. (2015) 14:456–70. doi: 10.1074/mcp.O114.046565 PMC4349969 25616866

[35] Lenoir O Flosseau K Ma FX Blondeau B Mai A Bassel-Duby R. Specific control of pancreatic endocrine beta- and delta-cell mass by class IIa histone deacetylases HDAC4, HDAC5, and HDAC9. Diabetes. (2011) 60:2861–71. doi: 10.2337/db11-0440 PMC3198089 21953612

[36] Raichur S Teh SH Ohwaki K Gaur V Long YC Hargreaves M. Histone deacetylase 5 regulates glucose uptake and insulin action in muscle cells. J Mol Endocrinol. (2012) 49:203–11. doi: 10.1530/JME-12-0095 22991226

[37] Klymenko O Brecklinghaus T Dille M Springer C de Wendt C Altenhofen D. Histone deacetylase 5 regulates interleukin 6 secretion and insulin action in skeletal muscle. Mol Metab. (2020) 42:101062. doi: 10.1016/j.molmet.2020.101062 32771698 PMC7481569

[38] Chang Y Sung E Ryu S Park YW Jang YM Park M. Insulin resistance is associated with gallstones even in non-obese, non-diabetic Korean men. J Korean Med Sci. (2008) 23:644–50. doi: 10.3346/jkms.2008.23.4.644 PMC2526403 18756051

[39] Kullmann S Valenta V Wagner R Tschritter O Machann J Haring HU. Brain insulin sensitivity is linked to adiposity and body fat distribution. Nat Commun. (2020) 11:1841. doi: 10.1038/s41467-020-15686-y 32296068 PMC7160151

[40] Qi D Wang J Zhao Y Yang Y Wang Y Wang H. JMJD1C-regulated lipid synthesis contributes to the maintenance of MLL-rearranged acute myeloid leukemia. Leuk Lymphoma. (2022) 63:2149–60. doi: 10.1080/10428194.2022.2068004 35468015

[41] Folseraas T Melum E Rausch P Juran BD Ellinghaus E Shiryaev A. Extended analysis of a genome-wide association study in primary sclerosing cholangitis detects multiple novel risk loci. J Hepatol. (2012) 57:366–75. doi: 10.1016/j.jhep.2012.03.031 PMC3399030 22521342

[42] Chambers JC Zhang W Sehmi J Li X Wass MN van der Harst P. Genome-wide association study identifies loci influencing concentrations of liver enzymes in plasma. Nat Genet. (2011) 43:1131–8. doi: 10.1038/ng.970 PMC3482372 22001757

[43] Maroni L Hohenester SD van de Graaf SFJ Tolenaars D van Lienden K Verheij J. Knockout of the primary sclerosing cholangitis-risk gene Fut2 causes liver disease in mice. Hepatology. (2017) 66:542–54. doi: 10.1002/hep.29029 28056490

[44] Komorniak N Martynova-Van Kley A Nalian A Wardziukiewicz W Skonieczna-Zydecka K Styburski D. Can the FUT 2 gene variant have an effect on the body weight of patients undergoing bariatric surgery?-preliminary, exploratory study. Nutrients. (2020) 12:2621. doi: 10.3390/nu12092621 32872099 PMC7551162

[45] Yan H Qin Q Chen J Yan S Li T Gao X. Gut microbiome alterations in patients with visceral obesity based on quantitative computed tomography. Front Cell Infect Microbiol. (2021) 11:823262. doi: 10.3389/fcimb.2021.823262 35127566 PMC8811355

[46] Jensen MD Ryan DH Apovian CM Ard JD Comuzzie AG Donato KA. 2013 AHA/ACC/TOS guideline for the management of overweight and obesity in adults: a report of the American College of Cardiology/American Heart Association Task Force on Practice Guidelines and The Obesity Society. Circulation. (2014) 129:S102–38. doi: 10.1161/01.cir.0000437739.71477.ee PMC5819889 24222017

