# Supplementary material for: Independent association of general and central adiposity with risk of gallstone disease: observational and genetic analyses
Source: Front Endocrinol (Lausanne). 2024 Mar 11;15:1367229. doi: 10.3389/fendo.2024.1367229 (PMC10961427; doi:10.3389/fendo.2024.1367229)
Supplement: Supplementary file 1 [file DataSheet_1.docx]

**Supporting Information**

**Independent association of general and central adiposity with risk of gallstone disease: Observational and genetic analyses**

Min Zhang et al.

Corresponding authors:

Xia Jiang, E-mail: [xiajiang@scu.edu.cn](mailto:xiajiang@scu.edu.cn)

Ben Zhang, E-mail: [ben.zhang@scu.edu.cn](mailto:ben.zhang@scu.edu.cn)

Contents:

Section A: Supporting Methods

Section B: **Supporting** Tables

Section C: **Supporting** Figures

List of **Supporting information:**

Section A: Supporting Methods

Section B: **Supporting** Tables

Supporting Table S1. Characteristics of studies investigating the association between central obesity and gallstone disease risk.

Supporting Table S2. Data sources, sample sizes, number of genetic instruments and *F*-statistics.

Supporting Table S3. STROBE-MR checklist of recommended items to address in reports of Mendelian randomization studies.

Supporting Table S4. Data sources of the factors included in multivariate Mendelian randomization.

Supporting Table S5. Baseline characteristics of study participants according to the incident of gallstone disease.

Supporting Table S6. Results of the sensitivity analyses in the cohort study.

Supporting Table S7. Results of subgroup analysis by sex in the cohort study.

Supporting Table S8. All pleiotropic loci underlying general obesity and gallstone disease identified by cross-trait meta-analysis.

Supporting Table S9. All pleiotropic loci underlying central obesity and gallstone disease identified by cross-trait meta-analysis.

Supporting Table S10. Other related phenotypes for all the pleiotropic loci by GWAS Catlog search.

Supporting Table S11. Functional annotation for the pleiotropic loci from cross-trait meta-analysis through HeploReg V4.1.

Section C: **Supporting** Figures

Figure S1. GTEx tissue enrichment analysis for expression of the shared genes underlying obesity and gallstone disease.

Figure S2. Box plot of betas in leave-one-out analysis in the Mendelian randomization.

Figure S3. Estimates of causal effect sizes for sex-specific Mendelian randomization.

Figure S4. Estimates of causal effect sizes for genetical predisposition to gallstone disease on obesity-related traits.

Section A: Supporting Methods

**Definitions of confounders in the UK Biobank cohort study**

We defined a diagnosis of the diseases based on the ICD-10 code or ICD-9 code: liver disease (ICD-10: K70, K73, K75; ICD-9:571), chronic kidney disease (ICD-10: N18; ICD-9: 582, 583, 585, 586, 587), T2DM (ICD-10: E11; ICD-9: 250), hypertension (ICD-10: I10; ICD-9: 401), Crohn's disease (ICD-10: K50; ICD-9: 555), ulcerative colitis (ICD-10: K51; ICD-9: 556), cholecystitis (ICD-10: K81; ICD-9: 5750, 5751), cholangitis (ICD-10: K830; ICD-9: 5761), pancreatitis (ICD-10: K85, K860, K861; ICD-9: 5770, 5771). We classified participants into five groups according to average household income before tax (household income) categories: less than £18,000, £18,000-30,999, £31,000-51,999, £52,000-100,000, and more than £100,000 ^1^. For physical activity measured by International Physical Activity Questionnaire (IPAQ), we classified participants into three categories: Low (less than 600 MET-minutes/week), moderate (600-1,800 MET-minutes/week), and High (more than 1,800 MET-minutes/week) ^2^. An individual was considered to have a sedentary behavior when the time spent for the either of the sedentary behaviors (watching television, using a computer for leisure, and driving) was bigger than 4 hours/day ^3^. Participants were classified as tea and coffee drinkers if they reported drinking more than one cup per day ^4^. We defined an ideal diet as an adequate intake of at least half of the 10 food groups as follows: increased consumption of vegetables (≥3 servings/day), fruits (≥3 servings/day), fish (≥2 servings/day), whole grains (≥21 servings/day), dairy (≥3 servings/day), dietary fiber (≥16.46 g/day) and saturated fat (≥30.08 g/day), and reduced consumption of processed meats (≤1 serving/week), sodium (salt added to food, ≤1 g/day) and total energy (≤8844.92 KJ/day) ^5-7^. Each variable was given a score of 0 or 1, with 1 representing the healthy diet component. If participants achieved the intake goal they were considered to have an adequate intake of the diet component. Adequate intake of at least half of all diet components (≥ 5 items) was considered as an ideal diet, less than half was considered a poor diet.

**Definition of novel pleiotropic SNPs identified by CPASSOC**

In our study, a “novel” SNP, which was prioritized by us and of particular interest to us, was defined as a shared SNP neither driven by any single trait nor in LD with index SNPs identified by single-trait GWAS(s) (LD *r*^2^ < 0.2). This means novel pleiotropic SNPs were independent (*r*^2^ < 0.20) of those previously reported genome-wide significant SNPs (of BMI/WHR/WHR_adj_BMI and GSD), and none of their neighboring SNPs within 1.0 Mb region reached *P* < 5×10^-8^ in the single-trait GWAS(s) (of BMI/WHR/WHR_adj_BMI and GSD).

**Mendelian randomization analysis**

Mendelian randomization (MR) uses genetic variants that are robustly associated with exposure as an instrument to assess causal relationships. MR depends on three key assumptions: (1) genetic instruments are significantly associated with exposure of interest; (2) genetic instruments are not related to any confounding factors of the exposure-outcome association; and (3) genetic instruments affect the outcome only via the exposure. For each exposure, the SNPs were selected following the three main assumptions. We included SNPs at a threshold of genome-wide significance (*p* < 5×10^-8^). We retained variants with the lowest *p*-value as independent instruments based on linkage disequilibrium (LD) as measured by *r*^2^ (*r*^2^ > 0.1 in the European 1000 Genome reference panel). To quantify the strength of instrumental variables, we further calculated *F*-statistics, and a threshold of 10 was typically recommended for MR analyses. Moreover, we performed several sensitivity analyses to assess the plausibility of the assumptions. First, in addition to IVW method, we adopted MR-Egger regression and weighted median methods to test the robustness of results under relaxed model assumptions. Second, we excluded pleiotropic IVs which were associated with potential confounders according to NHGRI-EBI GWAS Catalog. Third, we excluded palindromic IVs, in which alleles are represented by the same pair of letters on the forward and the backward strands. Fourth, we performed a leave-one-out analysis where one variant was removed at a time and IVW was performed based on the remaining variants. Finally, we applied MR-Pleiotropy Residual Sum and Outlier (MR-PRESSO) method to evaluate the presence of horizontal pleiotropy and to re-calculate causal effects after removing the detected outliers.

**Supporting Table S1.** Characteristics of studies investigating the association between central obesity and gallstone disease risk.

| **Study** | **PMID** | **Study_design** | **Country** | **Cases** | **Controls** | **Sex** | **Central_obesity Index** | **Comparism** | **Adjustment except for BMI** | | **Adjustment including BMI** | |
| --- | --- | --- | --- | --- | --- | --- | --- | --- | --- | --- | --- | --- |
|  |  |  |  |  |  |  |  |  | **Adjustment factors** | **OR (95%CI)** | **Adjustment factors** | **OR (95%CI)** |
| Kim,2021 | 34097775 | Cohort study | Korea | 67 | 5550 | Both | Waist circumference | M: >90, F: >85 cm | Age, sex, eGFR, GGT, smoking, alcohol, physical activity | 1.3 (0.98, 1.72) | NA | |
| Baratta,2021 | 33670445 | Case-control study | Italy | 102 | 512 | Both | Waist circumference | M: >102, F: >84 cm | No | 1.54 (0.94, 2.54) | NA | |
| Sheng,2020 | 33097057 | Case-control study | China | 835 | 835 | Both | Waist circumference | per unit | Age, gender, race, occupation, SBP, DBP, fasting blood glucose | 1.68 (1.57, 1.79) | NA | |
| Song,2020 | 32166900 | Cross-sectional study | China | 274 | 3735 | Both | Waist circumference | M: >90, F: >85 cm | No | 1.85 (1.44, 2.38) | Age, BP, lipids, FPG, 2hGlu, NFLD, MetS, family history, thyroid disease, BMI | 1.38 (0.86, 2.19) |
| Gu,2020 | 32011459 | Case-control study | China | 94 | 2194 | Both | Waist circumference | per unit | No | 1.02 (0.99, 1.04) | NA | |
| Dhamnetiya,2019 | 30911504 | Case-control study | India | 120 | 120 | Both | WHR | M: >0.90, F: >0.80 | Smoking, alcohol, physical exercise, diet, fats, proteins, and fruit | 1.54 (0.67, 3.56) | NA | |
| Kim,2019 | 30794560 | Cross-sectional study | Korea | 806 | 36495 | Male | Waist circumference | per unit | No | 1.19 (1, 1.42) | Thigh circumference, LDLC, HDLC, cholesterol, smoking, alcohol, BMI | 1.1 (0.88, 1.37) |
| Kim,2019 |  |  |  | 554 | 21544 | Female |  | per unit |  | 2.02 (1.67, 2.45) |  | 1.26 (0.98, 1.64) |
| Dhamnetiya,2018 | 30498320 | Case-control study | India | 120 | 120 | Both | WHR | high vs low | No | 2.64 (1.32, 5.26) | NA | |
| Liu,2018 | 29772027 | Cohort study | China | 3272 | 66900 | Male | Waist circumference | 84-91/<84 cm | Age, total cholesterol, triglycerides, smoker, drinking, diabetes, hypertension, physical activity | 1.27 (1.15, 1.4) | NA | |
| Liu,2018 |  |  |  | 3272 | 66900 | Male |  | >91/<84 cm |  | 1.53 (1.4, 1.68) |  |  |
| Liu,2018 |  |  |  | 1057 | 17718 | Female |  | 78-87/<78 cm |  | 1.44 (1.2, 1.73) |  |  |
| Liu,2018 |  |  |  | 1057 | 17718 | Female |  | >87/<78 cm |  | 1.85 (1.55, 2.22) |  |  |
| Kim,2017 | 28353587 | Cross-sectional study | China | 503 | 12075 | Female | Central obesity | WC>80cm or WHtR>0.5 | No | 1.26 (1.09, 1.46) | Age, BP, FPG, triglycerides, LDLC, chronic hepatitis B, BMI | 1.03 (0.81, 1.32) |
| Kim,2017 |  |  |  | 765 | 17201 | Male |  |  |  | 1.67 (1.4, 2.01) |  | 1.02 (0.85, 1.23) |
| Radmard,2015 | 26256899 | Cross-sectional study | Iran | 178 | 548 | Female | WHR | per 0.1 | Age, Wealth score, LDLC, HDLC, triglyceride, FBS, HbA2C, and fatty liver | 1.29 (1.05, 1.59) | NA | |
| Radmard,2015 |  |  |  | 88 | 680 | Male |  |  |  | 1.5 (1.08, 2.07) |  |  |
| Zhang,2014 | 25310024 | Cross-sectional study | China | 163 | 163 | Both | Waist circumference | per SD | Age, sex | 1.44 (1.01, 1.93) | NA | |
| Lin,2014 | 25070766 | Cross-sectional study | China | 741 | 11182 | Both | Waist circumference | per unit | Age, SBP, DBP, HDLC, TG, FPG | 1.01 (1, 1.02) | NA | |
| Breitfeld,2014 | 25054309 | Cross-sectional study | Germany | 189 | 833 | Both | Waist circumference | per unit | Age, sex | 1.04 (1.02, 1.06) | NA | |
| Shen,2014 | 24707283 | Cross-sectional study | China | 860 | 5651 | Both | Waist circumference | M: >90, F: >80 cm | Age, sex | 1.93 (1.64, 2.2) | NA | |
| Kim,2011 | 22016589 | Cross-sectional study | Korea | 173 | 3952 | Female | Waist circumference | >85 cm | Age | 1.43 (0.92, 2.22) | Additional adjust smoking, alcohol, glucose, lipid profiles, blood pressure, and HOMA-IR, BMI | 1.17 (0.69, 1.99) |
| Banim,2011 | 21623190 | Cohort study | UK | 95 | 11093 | Male | Waist circumference | 34-36/<34 inches | Age, physical activity and alcohol intake, hormone replacement therapy and parity (in female) | 1.95 (0.69, 5.55) | NA | |
| Banim,2011 |  |  |  |  |  |  |  | 36-38/<34 inches |  | 3.31 (1.27, 8.84) |  |  |
| Banim,2011 |  |  |  |  |  |  |  | 38-40/<34 inches |  | 2.66 (0.99, 7.21) |  |  |
| Banim,2011 |  |  |  |  |  |  |  | 40-42/<34 inches |  | 3.94 (1.45, 10.68) |  |  |
| Banim,2011 |  |  |  |  |  |  |  | >42/<34 inches |  | 3.4 (1.23, 9.37) |  |  |
| Banim,2011 |  |  |  | 201 | 12874 | Female |  | 28-30/<28 inches |  | 0.94 (0.49, 1.84) |  |  |
| Banim,2011 |  |  |  |  |  |  |  | 30-32/<28 inches |  | 1.6 (0.89, 2.88) |  |  |
| Banim,2011 |  |  |  |  |  |  |  | 32-34/<28 inches |  | 1.94 (1.07, 3.52) |  |  |
| Banim,2011 |  |  |  |  |  |  |  | 34-36/<28 inches |  | 2.88 (1.59, 5.21) |  |  |
| Banim,2011 |  |  |  |  |  |  |  | >36/<28 inches |  | 2.77 (1.56, 4.89) |  |  |
| Walcher,2009 | 19814821 | Cross-sectional study | Germany | 167 | 1962 | Both | Waist circumference | per cm | Age, gender, family history, vitamin C supplementation, smoking, alcohol, caffeine, vegetarian diet, lipid concentrations, physical activity, medication, and diabetes, BMI | 1.03 (1.01, 1.06) | NA | |
| Walcher,2009 |  |  |  |  |  |  | WHR | per unit |  | 21.36 (1.41, 322.54) |  |  |
| Hou,2009 | 19362277 | Case-control study | China | 8477 | 16954 | Female | WHR | 0.75-0.77/<0.75 | Age, education, family income, total caloric intake, total fat intake, number of pregnancies, and menopausal status | 1.1 (1, 1.3) | Additional adjust for BMI | 1.2 (1, 1.4) |
| Hou,2009 |  |  |  |  |  |  |  | 0.77-0.79/<0.75 |  | 1.4 (1.2, 1.5) |  | 1.4 (1.3, 1.6) |
| Hou,2009 |  |  |  |  |  |  |  | 0.79-0.80/<0.75 |  | 1.4 (1.2, 1.6) |  | 1.5 (1.3, 1.7) |
| Hou,2009 |  |  |  |  |  |  |  | 0.80-0.81/<0.75 |  | 1.4 (1.2, 1.5) |  | 1.5 (1.3, 1.7) |
| Hou,2009 |  |  |  |  |  |  |  | 0.81-0.83/<0.75 |  | 1.4 (1.3, 1.6) |  | 1.6 (1.4, 1.9) |
| Hou,2009 |  |  |  |  |  |  |  | 0.83-0.84/<0.75 |  | 1.5 (1.3, 1.7) |  | 1.7 (1.5, 1.9) |
| Hou,2009 |  |  |  |  |  |  |  | 0.84-0.86/<0.75 |  | 1.6 (1.4, 1.8) |  | 1.9 (1.7, 2.2) |
| Hou,2009 |  |  |  |  |  |  |  | 0.86-0.89/<0.75 |  | 1.7 (1.5, 1.9) |  | 2.1 (1.8, 2.3) |
| Hou,2009 |  |  |  |  |  |  |  | >0.89/<0.75 |  | 1.9 (1.7, 2.2) |  | 2.5 (2.2, 2.8) |
| Chang,2008 | 18756051 | Cross-sectional study | Korea | 440 | 19063 | Both | Waist circumference | >90 cm | Age, smoking, exercise, and alcohol | 1.29 (1.03, 1.61) | Additional adjust total cholesterol, glucose, and HOMA, BMI | 1.01 (1, 1.03) |
| Nervi,2006 | 16516330 | Case-control study | Chile | 299 | 582 | Both | Waist circumference | M: >102, F: >88 cm | Age, sex, hormones use and smoking | 1.9 (1.3, 2.8) | NA | |
| Tsai,2006 | 16478796 | Cohort study | US | 3179 | 39133 | Both | WHR | 0.70–0.74/<0.7 | Age, parity, oral contraceptive use, hormone replacement therapy, physical activity, smoking, thiazide diuretics, non-steroidal anti-inflammatory drugs, energy, alcohol, coffee, weight change | 1.11 (0.94, 1.31) | Additional adjust for BMI | 1.12 (0.95, 1.33) |
| Tsai,2006 |  |  |  |  |  |  |  | 0.74-0.78/<0.7 |  | 1.32 (1.13, 1.55) |  | 1.23 (1.05, 1.45) |
| Tsai,2006 |  |  |  |  |  |  |  | 0.78-0.82/<0.7 |  | 1.44 (1.22, 1.7) |  | 1.23 (1.04, 1.45) |
| Tsai,2006 |  |  |  |  |  |  |  | 0.82-0.86/<0.7 |  | 1.85 (1.56, 2.2) |  | 1.48 (1.25, 1.76) |
| Tsai,2006 |  |  |  |  |  |  |  | >0.86/<0.7 |  | 1.89 (1.59, 2.25) |  | 1.39 (1.16, 1.66) |
| Tsai,2006 |  |  |  |  |  |  | Waist circumference | 26-28/<26 inches |  | 1.13 (0.94, 1.36) |  | 1.09 (0.91, 1.31) |
| Tsai,2006 |  |  |  |  |  |  |  | 28-30/<26 inches |  | 1.44 (1.21, 1.72) |  | 1.27 (1.06, 1.52) |
| Tsai,2006 |  |  |  |  |  |  |  | 30-32/<26 inches |  | 1.92 (1.61, 2.29) |  | 1.51 (1.25, 1.82) |
| Tsai,2006 |  |  |  |  |  |  |  | 32-36/<26 inches |  | 2.48 (2.09, 2.94) |  | 1.68 (1.38, 2.03) |
| Tsai,2006 |  |  |  |  |  |  |  | >36/<26 inches |  | 3.12 (2.6, 3.74) |  | 1.72 (1.39, 2.13) |
| Mendez-Sanchez,2005 | 15786544 | Cross-sectional study | Mexico | 65 | 180 | Both | Waist circumference | M: >102,  F: >88 cm | Age, sex | 3.61 (1.95, 6.71) | NA | |
| Mendez-Sanchez,2005 |  |  |  |  |  |  | WHR | >0.85 |  | 2.2 (0.95, 5.12) |  |  |
| Mendez-Sanchez,2005 | 15784027 | Cross-sectional study | Mexico | 119 | 354 | Both | Waist circumference | M: >102,  F: >88 cm | No | 3.84 (2.11, 7) | Age, gender, BMI | 3.65 (1.95, 6.71) |
| Volzke,2005 | 15775677 | Cross-sectional study | Germany | 586 | 1564 | Female | WHR | per unit | Age, HDLC, LDLC, parity, physical activity, smoking | 7.39 (1.02, 53.51) | NA | |
| Tsai,2004 | 15213025 | Cohort study | US | 1117 | 28730 | Male | WHR | 0.89-0.92/<0.89 | Age, physical activity, dietary fiber intake, use of thiazide diuretics, use of nonsteroidal antiinflammatory drugs, smoking, alcohol, caffeine, and total energy intake | 1.28 (0.99, 1.66) | Additional adjust for BMI | 1.22 (0.94, 1.58) |
| Tsai,2004 |  |  |  |  |  |  |  | 0.92-0.94/<0.89 |  | 1.59 (1.23, 2.05) |  | 1.48 (1.15, 1.92) |
| Tsai,2004 |  |  |  |  |  |  |  | 0.94-0.96/<0.89 |  | 1.76 (1.37, 2.27) |  | 1.61 (1.25, 2.07) |
| Tsai,2004 |  |  |  |  |  |  |  | 0.96-0.99/<0.89 |  | 1.96 (1.53, 2.51) |  | 1.73 (1.35, 2.23) |
| Tsai,2004 |  |  |  |  |  |  |  | >0.99/<0.89 |  | 2.09 (1.65, 2.66) |  | 1.78 (1.38, 2.28) |
| Everhart,2002 | 12029637 | Cross-sectional study | USA | 1311 | 2045 | Female | Waist circumference | per cm | Age, examination site, Indian heritage, parity | 1.01 (0.99, 1.02) | NA | |
| Everhart,2002 |  |  |  | 369 | 1251 | Male |  | per cm |  | 1.02 (1, 1.05) |  |  |
| Boland,2002 | 11880221 | Cohort study | US | 179 | 5660 | Male | WHR | 0.93-0.96/<0.93 | Age, race, ARIC field center | 0.87 (0.5, 1.5) | Additional adjust for BMI | 0.89 (0.5, 1.5) |
| Boland,2002 |  |  |  |  |  |  |  | 0.96-0.99/<0.93 |  | 0.86 (0.5, 1.4) |  | 0.89 (0.5, 1.5) |
| Boland,2002 |  |  |  |  |  |  |  | >0.99/<0.93 |  | 1.17 (0.7, 1.9) |  | 1.14 (0.7, 2) |
| Boland,2002 |  |  |  | 370 | 6564 | Female |  | 0.83-0.89/<0.83 |  | 1.44 (1, 2.2) |  | 1.43 (0.9, 2.2) |
| Boland,2002 |  |  |  |  |  |  |  | 0.89-0.95/<0.83 |  | 2.16 (1.5, 3.2) |  | 1.98 (1.3, 3) |
| Boland,2002 |  |  |  |  |  |  |  | >0.95/<0.83 |  | 2 (1.3, 3) |  | 1.78 (1.1, 2.8) |
| Ruhl,2001 | 11679957 | Cross-sectional study | US | 1417 | 5857 | Female | WHR | 0.79-0.84/<0.79 | No | 1.5 (0.86, 2.6) | Age, ethnicity, total cholesterol, alcohol, diabetes, smoking, physical activity, and number of live births, BMI | 1.2 (0.65, 2.2) |
| Ruhl,2001 |  |  |  |  |  |  |  | 0.84-0.88/<0.79 |  | 2.8 (1.9, 4.2) |  | 1.6 (0.99, 2.5) |
| Ruhl,2001 |  |  |  |  |  |  |  | 0.88-0.94/<0.79 |  | 4.4 (2.9, 6.6) |  | 1.5 (0.92, 2.5) |
| Ruhl,2001 |  |  |  |  |  |  |  | >0.94/<0.79 |  | 5.9 (4, 8.9) |  | 1.7 (1, 2.8) |
| Ruhl,2001 |  |  |  |  |  | Male |  | 0.9-0.94/<0.9 |  | 1.4 (0.74, 2.7) |  | 0.89 (0.44, 1.8) |
| Ruhl,2001 |  |  |  |  |  |  |  | 0.94-0.97/<0.79 |  | 3 (1.7, 5.5) |  | 1.5 (0.74, 3.1) |
| Ruhl,2001 |  |  |  |  |  |  |  | 0.97-1.02/<0.79 |  | 4.9 (2.8, 8.6) |  | 1.6 (0.87, 3) |
| Ruhl,2001 |  |  |  |  |  |  |  | >1.02/<0.79 |  | 11.1 (6.3, 19.6) |  | 2.6 (1.2, 5.7) |
| Kodama,1999 | 10078858 | Cross-sectional study | Japan | 174 | 6906 | Male | WHR | 0.879–0.912/<0.879 | Hospital, rank, smoking, alcohol and glucose tolerance | 1 (0.6, 1.7) | Additional adjust for BMI | 0.9 (0.5, 1.5) |
| Kodama,1999 |  |  |  |  |  |  |  | 0.913–0.958/<0.879 |  | 1.5 (0.9, 2.5) |  | 1.3 (0.7, 2.3) |
| Kodama,1999 |  |  |  |  |  |  |  | >0.958/<0.879 |  | 1.9 (1, 3.6) |  | 1.7 (0.8, 3.4) |
| Kodama,1999 |  |  |  |  |  |  |  | 0.879–0.912/<0.879 |  | 1.4 (0.7, 2.8) |  | 1.2 (0.6, 2.6) |
| Kodama,1999 |  |  |  |  |  |  |  | 0.913–0.958/<0.879 |  | 1.8 (0.9, 3.6) |  | 1.4 (0.7, 3.1) |
| Kodama,1999 |  |  |  |  |  |  |  | >0.958/<0.879 |  | 3 (1.4, 6.7) |  | 2.1 (0.8, 5.3) |
| Villalpando,1997 | 8696071 | Cross-sectional study | Mexico | 19 | 920 | Male | WHR | >0.973 | Age, blood pressure, diabetes, triglycerides, total cholesterol, BMI | 1.43 (0.48, 5.12) | NA | |
| Villalpando,1997 |  |  |  | 124 | 1211 | Female |  | >0.973 |  | 1.46 (0.96, 2.22) |  |  |
| Kono,1995 | 7610355 | Case-control study | Japan | 41 | 2044 | Male | WHR | 0.88-0.90/<0.88 | Smoking, alcohol, physical exercise, and glucose tolerance | 1 (0.4, 2.7) | NA | |
| Kono,1995 |  |  |  |  |  |  |  | 0.91-0.93/<0.88 |  | 1.6 (0.6, 3.8) |  |  |
| Kono,1995 |  |  |  |  |  |  |  | >0.93/<0.88 |  | 1.6 (0.7, 4.2) |  |  |
| Kono,1995 |  |  |  |  |  |  |  | 0.88-0.90/<0.88 |  | 1 (0.3, 3) |  |  |
| Kono,1995 |  |  |  |  |  |  |  | 0.91-0.93/<0.88 |  | 1.2 (0.4, 3.5) |  |  |
| Kono,1995 |  |  |  |  |  |  |  | >0.93/<0.88 |  | 2.3 (0.8, 6.1) |  |  |

GSD: gallstone disease, BMI: body mass index, WHR: waist-to-hip ratio, WHtR: waist-to-height ratio, VAT: visceral adipose tissue thickness, SAT: subcutaneous adipose tissue thickness. M: Male, F: female, NA: Not Applicable

**Supporting Table S2.** Data sources, sample sizes, number of genetic instruments and *F*-statistics.

| **Traits** | **# IV** | **Sample size** | **R^2^** | **F-statistics** | **Author** | **Ethnicity** | **Journal** | **Year** | **Consortium or study** | **PMID** |
| --- | --- | --- | --- | --- | --- | --- | --- | --- | --- | --- |
| **Exposures** |  |  |  |  |  |  |  |  |  |  |
| **BMI** | 670 | 806,834 | 6.41% | 82.47 | Sara Pulit | Eur | Human Molecular Genetics | 2019 | UK Biobank and GIANT consortium | 30239722 |
| **WHR** | 316 | 697,734 | 3.38% | 77.22 | Sara Pulit | Eur | Human Molecular Genetics | 2019 | UK Biobank and GIANT consortium | 30239722 |
| **WHR_adj_BMI** | 346 | 694,649 | 4.37% | 91.71 | Sara Pulit | Eur | Human Molecular Genetics | 2019 | UK Biobank and GIANT consortium | 30239722 |
| **Outcome** |  |  |  |  |  |  |  |  |  |  |
| **GSD** | 75 | 43,639 cases/506,798 controls | 6.23% | 518.05 | Cameron Fairfield | Eur | Hepatology | 2022 | UK Biobank and FinnGen | 34651315 |

GSD: gallstone disease, BMI: body mass index, WHR: waist-to-hip ratio, WHR_adj_BMI: WHR adjusted for BMI, IV: instrumental variable.

**Supporting Table S3.** STROBE-MR checklist of recommended items to address in reports of Mendelian randomization studies

| **Item No.** | **Section** | **Checklist item** | **Page No.** | **Relevant text from manuscript** |
| --- | --- | --- | --- | --- |
| 1 | **TITLE and ABSTRACT** | Indicate Mendelian randomization (MR) as the study’s design in the title and/or the abstract if that is a main purpose of the study | 2 | Mendelian randomization confirmed a robust WHR-GSD causal relationship which attenuated yet remained significant after adjusting for BMI. |
|  | **INTRODUCTION** |  |  |  |
| 2 | **Background** | Explain the scientific background and rationale for the reported study. What is the exposure? Is a potential causal relationship between exposure and outcome plausible? Justify why MR is a helpful method to address the study question | 3 | Three Mendelian randomization (MR) have consistently quantified a 1.63-fold increased risk of GSD per-unit increment in BMI utilizing 97 BMI-associated IVs as well as 22,195 GSD cases and 472,022 non-cases. |
| 3 | **Objectives** | State specific objectives clearly, including pre-specified causal hypotheses (if any). State that MR is a method that, under specific assumptions, intends to estimate causal effects | 3 | In this study, we aimed to comprehensively investigate the role of obesity, both central and general, in the development of GSD through observational analysis and genome-wide cross-trait analysis. ...a two-sample MR to infer putative causal associations |
|  | **METHODS** |  |  |  |
| 4 | **Study design and data sources** | Present key elements of the study design early in the article. Consider including a table listing sources of data for all phases of the study. For each data source contributing to the analysis, describe the following: | 4, Table S2 | Data sources of BMI, WHR, and WHRadjBMI  Data sources of GSD  Details of data sources are listed in Supporting Table S2. |
|  | a) | Setting: Describe the study design and the underlying population, if possible. Describe the setting, locations, and relevant dates, including periods of recruitment, exposure, follow-up, and data collection, when available. | 4, Table S2 | The hitherto largest GWAS for BMI, WHR, and WHRadjBMI was performed by meta-analyzing data from the Genetic Investigation of ANthropometric Traits (GIANT) consortium and UKB, which comprised up to 806,834 individuals of European ancestry. |
|  | b) | Participants: Give the eligibility criteria, and the sources and methods of selection of participants. Report the sample size, and whether any power or sample size calculations were carried out prior to the main analysis | 4, Table S2 | This GWAS comprised 550,437 European participants, among which 43,639 individuals were diagnosed with GSD. |
|  | c) | Describe measurement, quality control and selection of genetic variants | Supporting Methods | For each exposure factor, the SNPs were filtered according to the three main assumptions of MR. We included SNPs at a threshold of genome-wide significance (*p* < 5×10^-8^). Then, we retained variants with the lowest *p*-value as independent instruments based on linkage disequilibrium (LD) as measured by *r*^2^ (when *r*^2^ > 0.1 in the European 1000 Genome reference panel). Finally, to quantify the strength of instrumental variables, we calculated *F*-statistics, and a threshold of the *F*-statistics > 10 was typically recommended for MR analyses. |
|  | d) | For each exposure, outcome, and other relevant variables, describe methods of assessment and diagnostic criteria for diseases | 4 | The diagnostic codes included ICD-10, ICD-9, OPCS4, OPCS3, Read codes (primary care), and UKB self-reported codes. |
|  | e) | Provide details of ethics committee approval and participant informed consent, if relevant | NA | NA |
| 5 | **Assumptions** | Explicitly state the three core IV assumptions for the main analysis (relevance, independence and exclusion restriction) as well assumptions for any additional or sensitivity analysis | 6,7,Supporting Methods | We performed several sensitivity analyses to validify MR results. |
| 6 | **Statistical methods: main analysis** | Describe statistical methods and statistics used | 6 | We applied an inverse-variance weighted (IVW) method as our primary MR approach. |
|  | a) | Describe how quantitative variables were handled in the analyses (i.e., scale, units, model) | NA |  |
|  | b) | Describe how genetic variants were handled in the analyses and, if applicable, how their weights were selected | 6 | We further used MR-Egger regression and weighted median methods to test the robustness of results under relaxed model assumptions. |
|  | c) | Describe the MR estimator (e.g. two-stage least squares, Wald ratio) and related statistics. Detail the included covariates and, in case of two-sample MR, whether the same covariate set was used for adjustment in the two samples | NA |  |
|  | d) | Explain how missing data were addressed | NA |  |
|  | e) | If applicable, indicate how multiple testing was addressed | 6 | We adopted a Bonferroni-corrected *P*<0.017 (0.05/3) as statistical significance. |
| 7 | **Assessment of assumptions** | Describe any methods or prior knowledge used to assess the assumptions or justify their validity | 6,7,Supporting Methods | Moreover, we performed several sensitivity analyses to assess the plausibility of the assumptions. |
| 8 | **Sensitivity analyses and additional analyses** | Describe any sensitivity analyses or additional analyses performed (e.g. comparison of effect estimates from different approaches, independent replication, bias analytic techniques, validation of instruments, simulations) | 11, 12 | We performed several sensitivity analyses to validify MR results. Fist,... |
| 9 | **Software and pre-registration** | | | |
|  | a) | Name statistical software and package(s), including version and settings used | 11 | We finally conducted a two-sample MR to make causal inferences, following STROBE-MR guidelines, by using the relevant packages in R (version 3.6.3). |
|  | b) | State whether the study protocol and details were pre-registered (as well as when and where) | NA |  |
|  | **RESULTS** |  |  |  |
| 10 | **Descriptive data** |  |  |  |
|  | a) | Report the numbers of individuals at each stage of included studies and reasons for exclusion. Consider use of a flow diagram | Table S2, Figure 1 |  |
|  | b) | Report summary statistics for phenotypic exposure(s), outcome(s), and other relevant variables (e.g. means, SDs, proportions) | Table S2 |  |
|  | c) | If the data sources include meta-analyses of previous studies, provide the assessments of heterogeneity across these studies | NA |  |
|  | d) | For two-sample MR:  i.  Provide justification of the similarity of the genetic variant-exposure associations between the exposure and outcome samples  ii.  Provide information on the number of individuals who overlap between the exposure and outcome studies | Table S2 |  |
| 11 | **Main results** |  |  |  |
|  | a) | Report the associations between genetic variant and exposure, and between genetic variant and outcome, preferably on an interpretable scale | 8 | The calculated R^2^ and *F*-statistic suggested robust IVs. |
|  | b) | Report MR estimates of the relationship between exposure and outcome, and the measures of uncertainty from the MR analysis, on an interpretable scale, such as odds ratio or relative risk per SD difference | 9 | Consistent with previous findings, genetically predicted BMI was associated with an increased risk of GSD (OR=1.67, 95%CI=1.56-1.78) using an updated number of IVs.  Genetically predicted WHR was associated with a significantly increased risk of GSD (OR=1.49, 95%CI=1.35-1.65) using 307 IVs. |
|  | c) | If relevant, consider translating estimates of relative risk into absolute risk for a meaningful time period | NA |  |
|  | d) | Consider plots to visualize results (e.g. forest plot, scatterplot of associations between genetic variants and outcome versus between genetic variants and exposure) | Figures 2, 3 |  |
| 12 | **Assessment of assumptions** | | | |
|  | a) | Report the assessment of the validity of the assumptions | 9 | The effect did not alter in weighted median or MR-Egger method… |
|  | b) | Report any additional statistics (e.g., assessments of heterogeneity across genetic variants, such as *I^2^*, Q statistic or E-value) | 9,  Table S2 | We observed no sign of horizontal pleiotropy (*P* for MR-Egger intercept=0.37)… |
| 13 | **Sensitivity analyses and additional analyses** | | | |
|  | a) | Report any sensitivity analyses to assess the robustness of the main results to violations of the assumptions | 9, Figure 2 | The association remained consistent across the weighted median method, MR-Egger approach, and sensitivity analyses removing palindromic IVs, pleiotropic IVs, or outliers (MR-PRESSO). |
|  | b) | Report results from other sensitivity analyses or additional analyses | 9 | Removing palindromic variants, pleiotropic variants or outliers (MR-PRESSO) yielded to a similar result and the leave-one-out analysis indicated no outlying SNP.  The association remained consistent across the weighted median method, MR-Egger approach, and sensitivity analyses removing palindromic IVs, pleiotropic IVs, or outliers (MR-PRESSO). |
|  | c) | Report any assessment of direction of causal relationship (e.g., bidirectional MR) | 9 | On the contrary, we did not observe … in the reverse-direction MR. |
|  | d) | When relevant, report and compare with estimates from non-MR analyses | NA |  |
|  | e) | Consider additional plots to visualize results (e.g., leave-one-out analyses) | 9 | The leave-one-out analysis also showed no outlying SNP. |
|  | **DISCUSSION** |  |  |  |
| 14 | **Key results** | Summarize key results with reference to study objectives | 9 | Consistent with previous findings, we confirmed a major etiological role of general obesity in the development of GSD. We further observed an independent effect of central obesity on GSD, |
| 15 | **Limitations** | Discuss limitations of the study, taking into account the validity of the IV assumptions, other sources of potential bias, and imprecision. Discuss both direction and magnitude of any potential bias and any efforts to address them | 11,12 | Third, the GWASs used in the genetic analysis came from different studies, and thus there may be heterogeneity and confounding effects. |
| 16 | **Interpretation** |  |  |  |
|  | a) | Meaning: Give a cautious overall interpretation of results in the context of their limitations and in comparison with other studies | 9,10 | In addition, as to the assessment of causal relationship, by using an almost doubled GSD cases (43,639 vs. 22,195) and significantly augmented number of IVs of BMI (>565 vs. 97) as compared to previous MRs, the statistical power in our MR was greatly improved. Importantly, after adjusting for WHR and other important confounders (such as diet, smoking, alcohol, CKD, and NFLD) which have not been done in previous MRs, the robust causal estimates also validated the reliability of the effect of general obesity on GSD |
|  | b) | Mechanism: Discuss underlying biological mechanisms that could drive a potential causal relationship between the investigated exposure and the outcome, and whether the gene-environment equivalence assumption is reasonable. Use causal language carefully, clarifying that IV estimates may provide causal effects only under certain assumptions | 10 | Indeed, a previous MR quantified the causal association between WHR and GSD and found a modest effect of 1.007 which might be of limited clinical relevance. That study, however, was hampered by a small number of IVs (n=34) and inadequate GSD cases (n=14,723). With a ten-times increased number of IVs as well as a greatly enlarged number of cases, we were able to quantify the effect of WHR with a decent statistical power. We were also able to reveal the independent role of WHR through controlling for important confounders including BMI and others. Our analysis demonstrates an independent effect of WHR alone on the pathological pathways leading to GSD while largely ruling out reverse causality. |
|  | c) | Clinical relevance: Discuss whether the results have clinical or public policy relevance, and to what extent they inform effect sizes of possible interventions | 11 | Our findings provide important clinical and public health implications… |
| 17 | **Generalizability** | Discuss the generalizability of the study results (a) to other populations, (b) across other exposure periods/timings, and (c) across other levels of exposure | 12 | Fourth, our findings were restricted to European population to control for population stratification, this might also limit the generalizability to other populations. |
| **OTHER INFORMATION** | | | | |
| 18 | **Funding** | Describe sources of funding and the role of funders in the present study and, if applicable, sources of funding for the databases and original study or studies on which the present study is based | 13 | This research was supported by the… |
| 19 | **Data and data sharing** | Provide the data used to perform all analyses or report where and how the data can be accessed, and reference these sources in the article. Provide the statistical code needed to reproduce the results in the article, or report whether the code is publicly accessible and if so, where | 12 | GWAS summary statistics for BMI, WHR, WHR_adj_BMI are publicly available from… |
| 20 | **Conflicts of Interest** | All authors should declare all potential conflicts of interest | 12 | Conflict of Interest Statement… |

This checklist is copyrighted by the Equator Network under the Creative Commons Attribution 3.0 Unported (CC BY 3.0) license ^8,9^.

**Supporting Table S4.** Data sources of the factors included in multivariate Mendelian randomization.

| **Factors** | **PMID** | **Author** | **Year** | **Journal** | **Sample size** | **Source** |
| --- | --- | --- | --- | --- | --- | --- |
| Overall diet^a^ | 35653391 | Pirastu | 2022 | PLoS Genetics | 445,779 | UKB |
| HDL-C | 34887591 | Graham | 2021 | Nature | 1,320,016 | GLGC and UKB |
| Triglyceride | 34887591 | Graham | 2021 | Nature | 1,320,016 | GLGC and UKB |
| SBP | 33230300 | Surendran | 2020 | Nature Genetics | 1,320,000 | Multiple |
| DBP | 33230300 | Surendran | 2020 | Nature Genetics | 1,320,000 | Multiple |
| Current Cigarettes | 30643251 | Liu | 2019 | Nature Genetics | 337,334 | European-ancestry |
| Alcohol_Per_Day | 30643251 | Liu | 2019 | Nature Genetics | 941,280 | European-ancestry |
| Childhood BMI | 33045005 | Vogelezang | 2020 | PLoS Genetics | 61,111 | European ancestry |
| Chronic kidney disease | 31152163 | Wuttke | 2019 | Nature Genetics | 41,395 cases/439,303 controls | CKDGen Consortium |
| Type 2 diabetes | 30297969 | Mahajan | 2018 | Nature Genetics | 74,124 cases/824,006 controls | DIAGRAM |
| Non-alcoholic fatty liver disease | 34535985 | Fairfield | 2022 | Hepatology Communications | 7,417 cases/405,829 controls | UKB |

^a^the overall diet was defined as 29 food items including fish, fruit, vegetable, and meat consumption. BMI: body mass index, HDL-C: high-density lipoprotein cholesterol, SBP: systolic blood pressure, DBP: diastolic blood pressure.

**Supporting Table S5.** Baseline characteristics of study participants according to the incident of gallstone disease.

| **Characteristic** | **Overall** | **Non-GSD** | **Incident GSD** |
| --- | --- | --- | --- |
|  | **(N=461336)** | **(N=446053)** | **(N=15283)** |
| Person-year (total) | 5526391.26 | 5428531.73 | 97859.53 |
| Person-year (follow_up) | 11.98 ± 2.21 | 12.17 ± 1.87 | 6.40 ± 3.49 |
| Age at recruitment (years) | 56.71 ± 8.03 | 56.66 ± 8.04 | 58.23 ± 7.76 |
| Body mass index (BMI, kg/m^2^) | 27.36 ± 4.75 | 27.28 ± 4.71 | 29.62 ± 5.37 |
| Waist-to-hip ratio | 0.87 ± 0.09 | 0.87 ± 0.09 | 0.89 ± 0.09 |
| Total cholesterol (mmol/L) | 5.71 (1.14) | 5.72 (1.14) | 5.64 (1.20) |
| HDL-C (mmol/L) | 1.45 (0.38) | 1.46 (0.39) | 1.36 (0.35) |
| LDL-C (mmol/L) | 3.57 (0.87) | 3.57 (0.87) | 3.56 (0.90) |
| Triglycerides (mmol/L) | 1.75 (1.02) | 1.74 (1.02) | 1.94 (1.04) |
| Total bilirubin (umol/L) | 9.13 (6.42-10.42) | 9.15 (6.46-10.44) | 9.02 (6.16-10.30) |
| **Gender** |  |  |  |
| Female | 249446 (54.07) | 239817 (53.76) | 53.76 (63.00) |
| Male | 211890 (45.93) | 206236 (46.24) | 5654 (37.00) |
| **Type 2 diabetes** |  |  |  |
| No | 453788 (98.36) | 439015 (98.42) | 14773 (96.66) |
| Yes | 7548 (1.64) | 7038 (1.58) | 510 (3.34) |
| **Hypertension** |  |  |  |
| No | 426873 (92.53) | 413586 (92.72) | 13287 (86.94) |
| Yes | 34463 (7.47) | 32467 (7.28) | 19996 (13.06) |
| **Liver disease** |  |  |  |
| No | 460589 (99.84) | 445374 (99.85) | 15215 (99.56) |
| Yes | 747 (0.16) | 679 (0.15) | 68 (0.44) |
| **Chronic kidney disease** |  |  |  |
| No | 460682 (99.86) | 445437 (99.86) | 15245 (99.75) |
| Yes | 654 (0.14) | 616 (0.14) | 38 (0.25) |
| **Current tobacco smoking** |  |  |  |
| No | 412905 (89.55) | 399343 (89.57) | 13562 (88.81) |
| Yes, on most or all days | 35849 (7.77) | 34507 (7.74) | 1342 (8.79) |
| Only occasionally | 12352 (2.68) | 11985 (2.69) | 367 (2.40) |
| **Average total household income before tax** | | | |
| <£18,000 | 87103 (22.07) | 83218 (21.78) | 3885 (31.00) |
| £18,000 to £30,999 | 100181 (25.38) | 96675 (25.30) | 3506 (27.97) |
| £31,000 to £51,999 | 103941 (26.34) | 100903 (26.40) | 3038 (24.24) |
| £52,000 to £100,000 | 81684 (20.70) | 79902 (20.91) | 1782 (14.22) |
| >£100,000 | 21762 (5.51) | 21440 (5.61) | 322 (2.57) |
| **Assessment center** |  |  |  |
| England | 407818 (88.4) | 394147 (88.36) | 13671 (89.45) |
| Scotland | 33870 (7.34) | 32694 (7.33) | 1176 (7.69) |
| Wales | 19648 (4.26) | 19212 (4.31) | 436 (2.85) |
| **Townsend deprivation index at recruitment** | | | |
| <-2.00 (least deprived) | 173074 (37.52) | 167956 (37.65) | 5118 (33.49) |
| –2.00 to 1.99 (average) | 220202 (47.73) | 212807 (47.71) | 7395 (48.39) |
| ≥2.00 (most deprived) | 68060 (14.75) | 65290 (14.64) | 2770 (18.12) |
| **Sedentary beheavior** |  |  |  |
| No | 287717 (62.72) | 279715 (63.06) | 8002 (52.79) |
| Yes | 170990 (37.28) | 163835 (36.94) | 7155 (47.21) |
| **Ideal diet** |  |  |  |
| No | 269338 (60.16) | 259459 (59.93) | 9879 (67.07) |
| Yes | 178339 (39.84) | 173488 (40.07) | 4851 (32.93) |
| **Tea consumption** |  |  |  |
| <1 cups/day | 80811 (17.52) | 78001 (17.49) | 2810 (18.39) |
| ≥1 cups/day | 380525 (82.48) | 368052 (82.51) | 12473 (81.61) |
| **Coffee consumption** |  |  |  |
| <1 cups/day | 97015 (21.03) | 93290 (20.91) | 3725 (24.37) |
| ≥1 cups/day | 364321 (78.97) | 352763 (79.09) | 11558 (75.63) |
| **Crohn's disease** |  |  |  |
| No | 460136 (99.74) | 444947 (99.75) | 15189 (99.38) |
| Yes | 1200 (0.26) | 1106 (0.25) | 94 (0.62) |
| **Ulcerative colitis** |  |  |  |
| No | 459030 (99.5) | 443865 (99.51) | 15165 (99.23) |
| Yes | 2306 (0.5) | 2188 (0.49) | 118 (0.77) |
| **Sleeve gastrectomy** |  |  |  |
| No | 461329 (100) | 446048 (100) | 15281 (99.99) |
| Yes | 7 (0) | 5 (0) | 2 (0.01) |
| **Cholecystitis** |  |  |  |
| No | 460233 (99.76) | 444987 (99.76) | 15246 (99.76) |
| Yes | 1103 (0.24) | 1066 (0.24) | 37 (0.24) |
| **Cholangitis** |  |  |  |
| No | 461248 (99.98) | 445972 (99.98) | 15276 (99.95) |
| Yes | 88 (0.02) | 81 (0.02) | 7 (0.05) |
| **Pancreatitis** |  |  |  |
| No | 460739 (99.87) | 445511 (99.88) | 15228 (99.64) |
| Yes | 597 (0.13) | 542 (0.12) | 55 (0.36) |
| **Alcohol intake frequency** |  |  |  |
| Daily or almost daily | 97897 (21.24) | 95707 (21.47) | 2190 (14.35) |
| Three or four times a week | 110578 (23.99) | 107777 (24.18) | 2801 (18.35) |
| Once or twice a week | 121455 (26.35) | 117401 (26.34) | 4054 (26.56) |
| One to three times a month | 51190 (11.10) | 49022 (11.00) | 2168 (14.20) |
| Special occasions only | 49354 (10.71) | 46858 (10.51) | 2496 (16.35) |
| Never | 30540 (6.62) | 28985 (6.50) | 1555 (10.19) |
| **IPAQ activity group** |  |  |  |
| Low | 60813 (16.69) | 58391 (16.53) | 2422 (21.54) |
| Moderate | 118685 (32.57) | 115015 (32.57) | 3670 (32.64) |
| High | 184892 (50.74) | 179739 (50.90) | 5153 (45.82) |

Baseline characteristics of UK Biobank participants were presented as mean ± standard deviation or median ± interquartile range for continuous variables, and as frequencies for categorical variables; IPAQ: International Physical Activity Questionnaire; GSD, gallstone disease; HDL-C: high-density lipoprotein cholesterol, LDL-C: low-density lipoprotein cholesterol.

**Supporting Table S6.** Results of the sensitivity analyses in the cohort study.

|  | **Sensitivity analysis 1**a | | **Sensitivity analysis 2**b | | **Sensitivity analysis 1+2**c | |
| --- | --- | --- | --- | --- | --- | --- |
|  | **HR (95%CI)**d | ***P*** | **HR (95%CI)**d | ***P*** | **HR (95%CI)**d | ***P*** |
| **General obesity** |  |  |  |  |  |  |
| Normal (BMI: 18.5-25 kg/m^2^) | 1.00 (ref) |  |  |  |  |  |
| Underweight (BMI: >18.5 kg/m^2^) | 0.68 (0.40, 1.15) | 0.1509 | 0.61 (0.36, 1.06) | 0.0801 | 0.66 (0.38, 1.14) | 0.134 |
| Overweight (BMI: 25-30 kg/m^2^) | 1.54 (1.44, 1.64) | <0.0001 | 1.57 (1.47, 1.67) | <0.0001 | 1.55 (1.45, 1.66) | <0.0001 |
| Obese class I (BMI: 30-35 kg/m^2^) | 2.04 (1.89, 2.20) | <0.0001 | 2.07 (1.92, 2.23) | <0.0001 | 2.07 (1.91, 2.23) | <0.0001 |
| Obese class II (BMI: 35-40 kg/m^2^) | 2.34 (2.12, 2.58) | <0.0001 | 2.40 (2.18, 2.64) | <0.0001 | 2.39 (2.16, 2.64) | <0.0001 |
| Obese class III (BMI: >40 kg/m^2^) | 2.32 (2.03, 2.66) | <0.0001 | 2.44 (2.14, 2.78) | <0.0001 | 2.37 (2.07, 2.72) | <0.0001 |
| P for trend | <0.0001 |  |  |  |  |  |
| BMI per 5 units | 1.28 (1.25, 1.31) | <0.0001 | 1.29 (1.26, 1.31) | <0.0001 | 1.28 (1.25, 1.31) | <0.0001 |
| **Central obesity** |  |  |  |  |  |  |
| Normal (WHR: Fe<0.85, Ma<0.9) | 1.00 (ref) |  |  |  |  |  |
| Central obesity (WHR: Fe>0.85, Ma>0.9) | 1.25 (1.18, 1.32) | <0.0001 | 1.25 (1.19, 1.32) | <0.0001 | 1.26 (1.19, 1.33) | <0.0001 |
| WHR_per_SD | 1.19 (1.15, 1.23) | <0.0001 | 1.18 (1.14, 1.22) | <0.0001 | 1.19 (1.15, 1.23) | <0.0001 |

^a^Sensitivity analysis that excluded participants with less than a year of follow-up or a diagnosis of GSD within a year after enrollment; ^b^Sensitivity analysis that excluded participants who underwent sleeve gastrectomy and diagnosed with Crohn's disease, ulcerative colitis, cholecystitis, cholangitis, and pancreatitis. ^c^Sensitivity analysis that excluded participants in Sensitivity analyses 1 and 2. ^d^Adjusted for age, sex, assessment center, the top 40 genetic principal components, total cholesterol, triglycerides, HDLC, T2DM, hypertension, current smoking, liver disease, chronic kidney disease, total bilirubin, income, drinking, physical activity, and use of anti-hyperlipidemia medication, anti-blood pressure medication, insulin medication use diet, sedentary behavior, tea consumption, coffee consumption, Townsend deprivation index and BMI or WHR.

**Supporting Table S7.** Results of subgroup analysis by sex in the cohort study.

|  | **Female** | | **Male** | | ***P* for Interaction** |
| --- | --- | --- | --- | --- | --- |
|  | **HR (95%CI)^a^** | ***P*** | **HR (95%CI)^a^** | ***P*** |  |
| **General obesity** |  |  |  |  | 0.001 |
| Normal (BMI: 18.5-25 kg/m^2^) | 1.00 (ref) |  |  |  |  |
| Underweight (BMI: >18.5 kg/m^2^) | 0.42 (0.20, 0.88) | 0.022 | 1.73 (0.82, 3.66) | 0.152 |  |
| Overweight (BMI: 25-30 kg/m^2^) | 1.78 (1.64, 1.93) | <0.0001 | 1.17 (1.05, 1.29) | 0.0042 |  |
| Obese class I (BMI: 30-35 kg/m^2^) | 2.39 (2.18, 2.63) | <0.0001 | 1.47 (1.30, 1.65) | <0.0001 |  |
| Obese class II (BMI: 35-40 kg/m^2^) | 2.79 (2.48, 3.14) | <0.0001 | 1.61 (1.37, 1.90) | <0.0001 |  |
| Obese class III (BMI: >40 kg/m^2^) | 2.76 (2.36, 3.22) | <0.0001 | 1.75 (1.38, 2.23) | <0.0001 |  |
| P for trend | <0.001 |  | <0.001 |  |  |
| BMI per 5 units | 1.32 (1.28, 1.35) | <0.0001 | 1.18 (1.13, 1.23) | <0.0001 | <0.001 |
| **Central obesity** |  |  |  |  |  |
| Normal (WHR: Fe<0.85, Ma<0.9) |  |  |  |  |  |
| Central obesity (WHR: Fe>0.85, Ma>0.9) | 1.24 (1.16, 1.32) | <0.0001 | 1.33 (1.21, 1.47) | <0.0001 | 0.210 |
| WHR_per_SD | 1.19 (1.14, 1.24) | <0.0001 | 1.20 (1.14, 1.27) | <0.0001 | 0.672 |

Adjusted for age, sex, assessment center, the top 40 genetic principal components, total cholesterol, triglycerides, HDLC, T2DM, hypertension, current smoking, liver disease, chronic kidney disease, total bilirubin, income, drinking, physical activity, and use of anti-hyperlipidemia medication, anti-blood pressure medication, insulin medication use diet, sedentary behavior, tea consumption, coffee consumption, Townsend deprivation index, Crohn's disease, ulcerative colitis, cholecystitis, cholangitis, pancreatitis and BMI or WHR.

**Supporting Table S8.** All pleiotropic loci underlying general obesity and gallstone disease identified by cross-trait meta-analysis.

| **SNP** | **CHR:BP** | **A1/A2** | **Obesity** | | **GSD** | | ***P*_CPASSOC_** | **ENCODE genes** | **Linear closest gene** | **Labled** |
| --- | --- | --- | --- | --- | --- | --- | --- | --- | --- | --- |
|  |  |  | **BETA** | ***P*** | **BETA** | ***P*** |  |  |  |  |
| **BMI and GSD** | |  |  |  |  |  |  |  |  |  |
| rs10136491 | 14:62539718 | A/G | -0.011 | 4.63E-08 | -0.047 | 4.92E-08 | 1.11E-13 | SYT16 | SYT16 | Known |
| rs10424365 | 19:19310527 | G/A | 0.012 | 5.87E-07 | 0.059 | 1.08E-06 | 2.26E-11 | RFXANK | RFXANK | Novel |
| rs1048365 | 7:100804430 | T/C | 0.013 | 2.22E-08 | -0.052 | 1.44E-06 | 5.29E-13 | AP1S1 | VGF | Single-trait-driven |
| rs10761785 | 10:65318766 | T/G | -0.013 | 3.47E-16 | 0.064 | 4.19E-17 | 2.43E-30 | REEP3 | REEP3 | Known |
| rs10858884 | 12:89893712 | G/A | 0.010 | 7.66E-08 | 0.055 | 3.44E-10 | 7.83E-15 | POC1B | POC1B | Single-trait-driven |
| rs10882884 | 10:99025611 | A/T | -0.009 | 7.55E-08 | -0.042 | 7.62E-08 | 2.77E-13 | ARHGAP19 | ARHGAP19-SLIT1 | LD-tagged |
| rs11012737 | 10:21849769 | A/G | 0.019 | 6.55E-25 | 0.048 | 3.54E-09 | 5.97E-34 | MLLT10 | MLLT10 | Known |
| rs11065363 | 12:121388498 | T/C | 0.011 | 6.32E-06 | 0.049 | 3.59E-06 | 9.83E-10 | HNF1A-AS1 | - | Novel |
| rs11672660 | 19:46180184 | T/C | -0.034 | 6.83E-60 | -0.060 | 1.81E-10 | 8.10E-72 | GIPR | GIPR | Known |
| rs11704728 | 22:18130209 | T/C | 0.013 | 2.31E-09 | 0.042 | 7.00E-06 | 1.33E-13 | BCL2L13 | BCL2L13 | Single-trait-driven |
| rs1260326 | 2:27730940 | C/T | 0.011 | 1.16E-10 | 0.079 | 1.22E-23 | 1.06E-26 | GCKR | GCKR | Known |
| rs12770588 | 10:21709301 | G/A | 0.014 | 1.14E-10 | 0.046 | 2.67E-06 | 1.88E-15 | RP11-275N1.1 | - | Single-trait-driven |
| rs12900395 | 15:77310345 | G/C | 0.009 | 9.08E-08 | 0.035 | 5.92E-06 | 8.26E-12 | PSTPIP1 | PSTPIP1 | Novel |
| rs12972158 | 19:46158473 | T/C | 0.013 | 1.23E-08 | 0.047 | 1.88E-06 | 3.23E-13 | Metazoa_SRP | RN7SL836P | Single-trait-driven |
| rs1374915 | 3:71668037 | C/T | -0.010 | 3.58E-07 | -0.038 | 1.03E-06 | 1.19E-11 | FOXP1 | - | Novel |
| rs147233090 | 15:44028047 | T/C | -0.029 | 5.49E-06 | 0.142 | 1.33E-07 | 9.50E-11 | - | CATSPER2P1 | Novel |
| rs17145750 | 7:73026378 | T/C | 0.012 | 7.70E-08 | 0.056 | 6.42E-08 | 2.52E-13 | MLXIPL | MLXIPL | LD-tagged |
| rs17716013 | 8:9178634 | G/T | -0.016 | 1.23E-18 | 0.039 | 4.09E-06 | 5.65E-24 | MIR597 | RP11-115J16.1 | Single-trait-driven |
| rs2457445 | 10:64781226 | A/G | -0.010 | 4.57E-08 | 0.043 | 8.38E-08 | 1.58E-13 | - | - | Single-trait-driven |
| rs2639998 | 3:141336708 | T/A | 0.011 | 3.92E-11 | 0.038 | 1.14E-06 | 2.71E-16 | RASA2 | RASA2 | Single-trait-driven |
| rs2798297 | 4:3064004 | A/G | 0.011 | 7.33E-08 | 0.037 | 3.15E-06 | 3.96E-12 | HTT-AS1 | HTT-AS | LD-tagged |
| rs2954021 | 8:126482077 | G/A | 0.011 | 2.41E-10 | 0.049 | 1.32E-10 | 2.38E-18 | TRIB1 | RP11-136O12.2 | Known |
| rs34811474 | 4:25408838 | A/G | -0.029 | 8.50E-38 | -0.041 | 6.78E-06 | 3.81E-43 | ANAPC4 | ANAPC4 | Single-trait-driven |
| rs362307 | 4:3241845 | T/C | 0.024 | 1.67E-10 | 0.065 | 6.17E-06 | 6.00E-15 | HTT | HTT | Single-trait-driven |
| rs3744405 | 17:7193255 | A/G | 0.008 | 4.11E-06 | 0.035 | 3.47E-06 | 5.62E-10 | YBX2 | YBX2 | Novel |
| rs3764002 | 12:108618630 | T/C | -0.011 | 2.53E-08 | -0.038 | 9.83E-06 | 2.76E-12 | WSCD2 | WSCD2 | Single-trait-driven |
| rs451643 | 5:176511432 | T/G | 0.009 | 4.33E-06 | -0.040 | 1.23E-06 | 2.98E-10 | FGFR4 | FGFR4 | LD-tagged |
| rs4886838 | 15:77158170 | T/C | 0.010 | 6.14E-06 | 0.046 | 2.30E-06 | 7.02E-10 | SCAPER | SCAPER | Novel |
| rs6001872 | 22:40703245 | G/A | -0.016 | 5.63E-19 | 0.056 | 1.37E-12 | 2.22E-30 | TNRC6B | TNRC6B | Known |
| rs6486122 | 11:13361524 | T/C | 0.011 | 2.61E-09 | 0.037 | 3.74E-06 | 9.08E-14 | ARNTL | ARNTL | Single-trait-driven |
| rs6544597 | 2:43013593 | G/T | 0.009 | 3.54E-06 | 0.045 | 2.22E-07 | 7.46E-11 | HAAO | HAAO | Novel |
| rs6744393 | 2:27750139 | T/C | 0.012 | 1.65E-06 | 0.052 | 2.42E-07 | 2.96E-11 | GCKR | GCKR | LD-tagged |
| rs6744550 | 2:60285544 | C/G | 0.013 | 5.46E-11 | 0.038 | 8.85E-07 | 3.19E-16 | AC007381.3 | RP11-444A22.1 | Single-trait-driven |
| rs715 | 2:211543055 | C/T | 0.016 | 1.34E-16 | -0.047 | 1.28E-08 | 3.19E-24 | CPS1 | CPS1 | Known |
| rs7200764 | 16:69548788 | C/T | -0.017 | 2.39E-13 | -0.051 | 1.46E-06 | 1.07E-18 | CYB5B | - | Single-trait-driven |
| rs7412 | 19:45412079 | T/C | 0.020 | 7.03E-09 | 0.088 | 8.18E-10 | 6.14E-16 | APOE | APOE | Known |
| rs7550711 | 1:110082886 | T/C | 0.066 | 9.11E-39 | 0.119 | 3.47E-09 | 1.35E-48 | GPR61 | GPR61 | Known |
| rs76637437 | 10:65079361 | C/T | -0.015 | 9.35E-07 | 0.062 | 1.10E-06 | 4.07E-11 | JMJD1C | JMJD1C | Novel |
| rs786420 | 2:44719893 | T/C | 0.014 | 2.27E-12 | 0.055 | 4.53E-10 | 1.79E-20 | CAMKMT | CAMKMT | Known |
| rs836509 | 7:6457655 | C/T | 0.009 | 1.85E-07 | 0.040 | 1.28E-07 | 1.22E-12 | DAGLB | DAGLB | LD-tagged |
| rs889398 | 16:69556715 | T/C | -0.020 | 3.23E-32 | -0.041 | 1.22E-07 | 6.48E-40 | NFAT5 | - | Single-trait-driven |
| rs9571577 | 13:66937503 | G/A | 0.009 | 2.70E-06 | 0.036 | 1.69E-06 | 2.04E-10 | PCDH9 | PCDH9 | Novel |
| rs9625962 | 22:44326272 | C/T | -0.013 | 1.80E-06 | -0.050 | 9.29E-07 | 8.17E-11 | PNPLA3 | PNPLA3 | Novel |
| rs970635 | 14:40123072 | C/T | 0.012 | 1.86E-08 | 0.043 | 5.32E-06 | 1.18E-12 | RP11-111A21.1 | - | Single-trait-driven |

A1/A2: effect allele/other allele, BMI: body mass index, GSD: gallstone disease, WHR: waist-to-hip ratio, WHRadjBMI: waist-to-hip ratio adjusted for body mass index. Linear closest genes of index SNPs were mapped by using VEP; GENCODE genes of index SNPs were mapped by HeploReg V4.1.

**Supporting Table S9.** All pleiotropic loci underlying central obesity and gallstone disease identified by cross-trait meta-analysis.

| **SNP** | **CHR:BP** | **A1/A2** | **Obesity** | | **GSD** | | ***P*_CPASSOC_** | **ENCODE genes** | **Linear closest gene** | **Labled** |
| --- | --- | --- | --- | --- | --- | --- | --- | --- | --- | --- |
|  |  |  | **BETA** | ***P*** | **BETA** | ***P*** |  |  |  |  |
| **WHR and GSD** | |  |  |  |  |  |  |  |  |  |
| rs10761785 | 10:65318766 | T/G | -0.015 | 6.43E-18 | 0.064 | 4.19E-17 | 1.40E-33 | REEP3 | REEP3 | Known |
| rs10822180 | 10:65310105 | A/G | -0.013 | 1.43E-07 | 0.058 | 1.37E-08 | 6.25E-14 | REEP3 | REEP3 | Single-trait-driven |
| rs10995543 | 10:65239547 | C/T | 0.014 | 2.36E-08 | -0.049 | 9.70E-06 | 1.60E-12 | JMJD1C | - | Single-trait-driven |
| rs11012737 | 10:21849769 | A/G | 0.015 | 3.82E-14 | 0.048 | 3.54E-09 | 3.07E-22 | MLLT10 | MLLT10 | Known |
| rs13429408 | 2:219142860 | A/C | -0.011 | 3.38E-09 | 0.050 | 1.89E-10 | 2.13E-17 | PNKD | TMBIM1 | Known |
| rs1558902 | 16:53803574 | A/T | 0.040 | 3.99E-118 | 0.035 | 7.22E-06 | 9.68E-128 | FTO | FTO | Single-trait-driven |
| rs1800437 | 19:46181392 | C/G | -0.021 | 6.23E-22 | -0.060 | 1.87E-10 | 3.85E-32 | GIPR | GIPR | Known |
| rs228757 | 17:42164885 | C/G | -0.010 | 4.24E-07 | 0.044 | 6.45E-07 | 4.72E-12 | HDAC5 | HDAC5 | Novel |
| rs2457445 | 10:64781226 | A/G | -0.010 | 1.74E-07 | 0.043 | 8.38E-08 | 3.21E-13 | - | - | Novel |
| rs2686189 | 8:11655229 | T/C | -0.009 | 8.61E-06 | -0.038 | 3.36E-06 | 6.69E-10 | FDFT1 | FDFT1 | Novel |
| rs2980856 | 8:126476379 | G/C | -0.008 | 6.44E-07 | 0.046 | 2.14E-09 | 1.12E-13 | TRIB1 | RP11-136O12.2 | Single-trait-driven |
| rs3121419 | 4:3232257 | T/C | -0.013 | 1.59E-11 | -0.038 | 2.66E-06 | 1.44E-16 | HTT | HTT | Single-trait-driven |
| rs36120341 | 19:46224971 | T/C | 0.010 | 1.16E-06 | 0.076 | 2.72E-21 | 5.55E-22 | FBXO46 | FBXO46 | Single-trait-driven |
| rs3764002 | 12:108618630 | T/C | -0.021 | 3.91E-27 | -0.038 | 9.83E-06 | 1.04E-31 | WSCD2 | WSCD2 | Single-trait-driven |
| rs429358 | 19:45411941 | C/T | -0.035 | 4.21E-37 | -0.056 | 1.02E-07 | 2.94E-44 | APOE | APOE | Single-trait-driven |
| rs55747707 | 7:73037366 | A/G | -0.015 | 3.40E-11 | 0.055 | 4.99E-09 | 1.08E-18 | MLXIPL | MLXIPL | Known |
| rs6486122 | 11:13361524 | T/C | 0.011 | 1.02E-08 | 0.037 | 3.74E-06 | 2.61E-13 | ARNTL | ARNTL | Single-trait-driven |
| rs6889220 | 5:176693888 | A/G | 0.014 | 4.28E-07 | 0.051 | 6.79E-06 | 3.25E-11 | NSD1 | NSD1 | Novel |
| rs702484 | 7:6431933 | G/C | 0.010 | 5.27E-07 | 0.051 | 3.03E-10 | 2.15E-14 | RAC1 | RAC1 | Single-trait-driven |
| rs7193778 | 16:69563890 | T/C | -0.017 | 7.87E-11 | -0.051 | 1.80E-06 | 5.73E-16 | NFAT5 | - | Single-trait-driven |
| rs733381 | 22:40669648 | G/A | -0.012 | 2.82E-09 | 0.046 | 2.87E-07 | 6.29E-15 | TNRC6B | TNRC6B | Single-trait-driven |
| rs73942938 | 19:46162346 | C/T | 0.018 | 1.56E-13 | 0.048 | 1.39E-06 | 5.02E-19 | Metazoa_SRP | - | Single-trait-driven |
| rs7550711 | 1:110082886 | T/C | 0.030 | 1.18E-08 | 0.119 | 3.47E-09 | 9.57E-16 | GPR61 | GPR61 | Known |
| rs889398 | 16:69556715 | T/C | -0.017 | 1.99E-23 | -0.041 | 1.22E-07 | 1.46E-30 | NFAT5 | - | Single-trait-driven |
| **WHRadjBMI and GSD** | |  |  |  |  |  |  |  |  |  |
| rs1047891 | 2:211540507 | A/C | -0.009 | 1.08E-06 | -0.047 | 9.28E-09 | 5.73E-13 | CPS1 | CPS1 | Single-trait-driven |
| rs112875651 | 8:126506694 | A/G | -0.019 | 1.14E-19 | 0.047 | 9.91E-10 | 6.70E-29 | TRIB1 | RP11-136O12.2 | Known |
| rs13280055 | 8:11522353 | A/G | 0.017 | 1.01E-08 | 0.089 | 3.85E-15 | 3.82E-20 | GATA4 | - | Known |
| rs13429408 | 2:219142860 | A/C | -0.010 | 8.75E-08 | 0.050 | 1.89E-10 | 1.31E-15 | PNKD | TMBIM1 | Single-trait-driven |
| rs1883016 | 8:11629637 | A/G | 0.015 | 1.74E-10 | 0.059 | 5.20E-11 | 1.76E-19 | NEIL2 | NEIL2 | Known |
| rs2256249 | 8:11494527 | T/G | -0.013 | 3.56E-10 | -0.037 | 1.50E-06 | 2.50E-15 | GATA4 | - | Single-trait-driven |
| rs3121419 | 4:3232257 | T/C | -0.011 | 8.54E-09 | -0.038 | 2.66E-06 | 1.51E-13 | HTT | HTT | Single-trait-driven |
| rs36120341 | 19:46224971 | T/C | 0.010 | 3.43E-06 | 0.076 | 2.72E-21 | 6.51E-22 | FBXO46 | FBXO46 | Single-trait-driven |
| rs3744405 | 17:7193255 | A/G | -0.010 | 4.54E-08 | 0.035 | 3.47E-06 | 1.29E-12 | YBX2 | YBX2 | Single-trait-driven |
| rs3764002 | 12:108618630 | T/C | -0.018 | 1.86E-19 | -0.038 | 9.83E-06 | 3.86E-24 | WSCD2 | WSCD2 | Single-trait-driven |
| rs3784924 | 16:11644842 | G/A | 0.011 | 9.28E-09 | 0.062 | 2.45E-14 | 1.19E-19 | LITAF | LITAF | Known |
| rs3828944 | 7:6487156 | C/T | -0.010 | 2.54E-06 | -0.062 | 2.39E-12 | 6.81E-15 | DAGLB | DAGLB | Single-trait-driven |
| rs429358 | 19:45411941 | C/T | -0.026 | 2.29E-21 | -0.056 | 1.02E-07 | 1.72E-28 | APOE | APOE | Single-trait-driven |
| rs4454603 | 10:65012750 | T/C | -0.009 | 2.38E-07 | 0.067 | 7.32E-19 | 1.76E-20 | JMJD1C | JMJD1C | Single-trait-driven |
| rs451643 | 5:176511432 | T/G | -0.013 | 2.06E-11 | -0.040 | 1.23E-06 | 8.82E-17 | FGFR4 | FGFR4 | Single-trait-driven |
| rs55666908 | 10:65273534 | A/G | 0.014 | 3.85E-06 | -0.056 | 4.71E-06 | 3.03E-10 | REEP3 | - | Novel |
| rs55747707 | 7:73037366 | A/G | -0.024 | 3.05E-25 | 0.055 | 4.99E-09 | 5.05E-34 | MLXIPL | MLXIPL | Known |
| rs583484 | 8:9794369 | C/T | 0.013 | 2.20E-09 | 0.043 | 7.75E-06 | 8.81E-14 | snoU13 | - | Single-trait-driven |
| rs601338 | 19:49206674 | A/G | 0.010 | 1.25E-07 | 0.090 | 4.93E-32 | 1.63E-33 | FUT2 | FUT2 | Single-trait-driven |
| rs62130338 | 19:49162501 | A/G | 0.011 | 1.06E-06 | 0.040 | 5.31E-06 | 7.17E-11 | NTN5 | NTN5 | LD-tagged |
| rs6601599 | 8:11402347 | A/G | -0.016 | 2.04E-15 | -0.039 | 4.46E-07 | 1.50E-21 | BLK | BLK | Single-trait-driven |
| rs7012814 | 8:9173358 | A/G | -0.015 | 2.99E-13 | -0.044 | 6.62E-09 | 5.63E-21 | MIR597,TNKS | RP11-115J16.1 | Known |
| rs73942938 | 19:46162346 | C/T | 0.015 | 4.46E-09 | 0.048 | 1.39E-06 | 4.05E-14 | Metazoa_SRP | - | Single-trait-driven |
| rs889398 | 16:69556715 | T/C | -0.009 | 2.97E-07 | -0.041 | 1.22E-07 | 7.70E-13 | NFAT5 | - | LD-tagged |

A1/A2: effect allele/other allele, BMI: body mass index, GSD: gallstone disease, WHR: waist-to-hip ratio, WHRadjBMI: waist-to-hip ratio adjusted for body mass index. Linear closest genes of index SNPs were mapped by using VEP; GENCODE genes of index SNPs were mapped by HeploReg V4.1.

**Supporting Table S10.** Other related phenotypes for all the pleiotropic loci by GWAS Catlog search.

| **SNP** | **PCPASSOC** | **ENCODE genes** | **Class** | **GWAS_Catlog_Searcha** |
| --- | --- | --- | --- | --- |
| **BMI and GSD** | | | | |
| rs10136491 | 1.11E-13 | SYT16 | Known | - |
| rs10424365 | 2.26E-11 | RFXANK | Novel | - |
| rs1048365 | 5.29E-13 | AP1S1 | Single-trait-driven | - |
| rs10761785 | 2.43E-30 | REEP3 | Known | Plasma omega-6 polyunsaturated fatty acid levels |
| rs10858884 | 7.83E-15 | POC1B | Single-trait-driven | - |
| rs10882884 | 2.77E-13 | ARHGAP19 | LD-tagged | - |
| rs11012737 | 5.97E-34 | MLLT10 | Known | Endometriosis or asthma |
| rs11065363 | 9.83E-10 | HNF1A-AS1 | Novel | - |
| rs11672660 | 8.10E-72 | GIPR | Known | Blood urea nitrogen levels, Hip circumference |
| rs11704728 | 1.33E-13 | BCL2L13 | Single-trait-driven | - |
| rs1260326 | 1.06E-26 | GCKR | Known | Serum metabolite levels, T2D, cholesterol… |
| rs12770588 | 1.88E-15 | RP11-275N1.1 | Single-trait-driven | - |
| rs12900395 | 8.26E-12 | PSTPIP1 | Novel | - |
| rs12972158 | 3.23E-13 | Metazoa_SRP | Single-trait-driven | - |
| rs1374915 | 1.19E-11 | FOXP1 | Novel | - |
| rs147233090 | 9.50E-11 | 1.6kb 5' of U6 | Novel | Hemoglobin levels, Triglyceride levels, Calcium levels… |
| rs17145750 | 2.52E-13 | MLXIPL | LD-tagged | Sex hormone-binding globulin levels, Phosphatidylcholine, Sphingomyelin… |
| rs17716013 | 5.65E-24 | MIR597,TNKS | Single-trait-driven | - |
| rs2457445 | 1.58E-13 | 89kb 5' of U6 | Single-trait-driven | - |
| rs2639998 | 2.71E-16 | RASA2 | Single-trait-driven | - |
| rs2798297 | 3.96E-12 | HTT-AS1 | LD-tagged | Triglycerides, Sex hormone-binding globulin levels, Alanine aminotransferase levels… |
| rs2954021 | 2.38E-18 | TRIB1 | Known | Triglyceride levels, cholesterol levels, Alanine aminotransferase levels… |
| rs34811474 | 3.81E-43 | ANAPC4 | Single-trait-driven | Heel bone mineral density, Educational attainment, Osteoarthritis … |
| rs362307 | 6.00E-15 | HTT | Single-trait-driven | Type 2 diabetes, Predicted visceral adipose tissue, General cognitive ability … |
| rs3744405 | 5.62E-10 | YBX2 | Novel | - |
| rs3764002 | 2.76E-12 | WSCD2 | Single-trait-driven | Type 2 diabetes, Appendicular lean mass, Sex hormone-binding globulin levels… |
| rs451643 | 2.98E-10 | FGFR4 | LD-tagged | Appendicular lean mass |
| rs4886838 | 7.02E-10 | SCAPER | Novel | - |
| rs6001872 | 2.22E-30 | TNRC6B | Known | Body size, Diverticular disease, Impaired insulin sensitivity |
| rs6486122 | 9.08E-14 | ARNTL | Single-trait-driven | C-reactive protein levels, Triglyceride levels, Plasminogen activator inhibitor type 1 levels… |
| rs6544597 | 7.46E-11 | HAAO | Novel | - |
| rs6744393 | 2.96E-11 | GCKR | LD-tagged | Eosinophil counts |
| rs6744550 | 3.19E-16 | AC007381.3 | Single-trait-driven | - |
| rs715 | 3.19E-24 | CPS1 | Known | Blood metabolite levels, Glycine levels, Fibrinogen levels… |
| rs7200764 | 1.07E-18 | CYB5B | Single-trait-driven | - |
| rs7412 | 6.14E-16 | APOE | Known | Blood protein levels, cholesterol levels, Coronary artery disease. |
| rs7550711 | 1.35E-48 | GPR61 | Known | WC, Predicted visceral adipose tissue, Stearic acid (18:0) levels |
| rs76637437 | 4.07E-11 | JMJD1C | Novel | - |
| rs786420 | 1.79E-20 | CAMKMT | Known | - |
| rs836509 | 1.22E-12 | DAGLB | LD-tagged | - |
| rs889398 | 6.48E-40 | NFAT5 | Single-trait-driven | Lifetime smoking index, Smoking status |
| rs9571577 | 2.04E-10 | PCDH9 | Novel | - |
| rs9625962 | 8.17E-11 | PNPLA3 | Novel | Triacylglycerol |
| rs970635 | 1.18E-12 | RP11-111A21.1 | Single-trait-driven | - |
| **WHR and GSD** | | | | |
| rs10761785 | 1.40E-33 | REEP3 | Known | Plasma omega-6 polyunsaturated fatty acid levels |
| rs10822180 | 6.25E-14 | REEP3 | Single-trait-driven | - |
| rs10995543 | 1.60E-12 | JMJD1C | Single-trait-driven | - |
| rs11012737 | 3.07E-22 | MLLT10 | Known | Endometriosis or asthma |
| rs13429408 | 2.13E-17 | PNKD | Known | - |
| rs1558902 | 9.68E-128 | FTO | Single-trait-driven | Hip circumference, Alcohol consumption, Menarche... |
| rs1800437 | 3.85E-32 | GIPR | Known | Type 2 diabetes, Insulin levels, Pulse pressure… |
| rs228757 | 4.72E-12 | HDAC5 | Novel | - |
| rs2457445 | 3.21E-13 | 89kb 5' of U6 | Novel | - |
| rs2686189 | 6.69E-10 | FDFT1 | Novel | - |
| rs2980856 | 1.12E-13 | TRIB1 | Single-trait-driven | - |
| rs3121419 | 1.44E-16 | HTT | Single-trait-driven | - |
| rs36120341 | 5.55E-22 | FBXO46 | Single-trait-driven | Eosinophil counts |
| rs3764002 | 1.04E-31 | WSCD2 | Single-trait-driven | Type 2 diabetes, Appendicular lean mass, Sex hormone-binding globulin levels… |
| rs429358 | 2.94E-44 | APOE | Single-trait-driven | cholesterol levels, Type 2 diabetes, Longevity…. |
| rs55747707 | 1.08E-18 | MLXIPL | Known | cholesterol levels, Total fatty acid levels, Serum albumin levels… |
| rs6486122 | 2.61E-13 | ARNTL | Single-trait-driven | C-reactive protein levels, Triglyceride levels, Plasminogen activator inhibitor type 1 levels… |
| rs6889220 | 3.25E-11 | NSD1 | Novel | - |
| rs702484 | 2.15E-14 | RAC1 | Single-trait-driven | - |
| rs7193778 | 5.73E-16 | NFAT5 | Single-trait-driven | Serum uric acid levels, Urate levels |
| rs733381 | 6.29E-15 | TNRC6B | Single-trait-driven | - |
| rs73942938 | 5.02E-19 | Metazoa_SRP | Single-trait-driven | - |
| rs7550711 | 9.57E-16 | GPR61 | Known | Waist circumference, Predicted visceral adipose tissue, Stearic acid (18:0) levels |
| rs889398 | 1.46E-30 | NFAT5 | Single-trait-driven | Lifetime smoking index, Smoking status |
| **WHRadjBMI and GSD** | | | | |
| rs1047891 | 5.73E-13 | CPS1 | Single-trait-driven | Serum metabolite levels,Urinary metabolite modules , Sex hormone-binding globulin levels ... |
| rs112875651 | 6.70E-29 | TRIB1 | Known | cholesterol, Phospholipid levels, Triglycerides... |
| rs13280055 | 3.82E-20 | GATA4 | Known | Sex hormone-binding globulin levels |
| rs13429408 | 1.31E-15 | PNKD | Single-trait-driven | - |
| rs1883016 | 1.76E-19 | NEIL2 | Known | Cholecystitis |
| rs2256249 | 2.50E-15 | GATA4 | Single-trait-driven | Waist circumference |
| rs3121419 | 1.51E-13 | HTT | Single-trait-driven | - |
| rs36120341 | 6.51E-22 | FBXO46 | Single-trait-driven | Eosinophil counts |
| rs3744405 | 1.29E-12 | YBX2 | Single-trait-driven | - |
| rs3764002 | 3.86E-24 | WSCD2 | Single-trait-driven | Type 2 diabetes, Appendicular lean mass, Sex hormone-binding globulin levels… |
| rs3784924 | 1.19E-19 | LITAF | Known | Liver enzyme levels |
| rs3828944 | 6.81E-15 | DAGLB | Single-trait-driven | - |
| rs429358 | 1.72E-28 | APOE | Single-trait-driven | cholesterol levels, Type 2 diabetes, Longevity…. |
| rs4454603 | 1.76E-20 | JMJD1C | Single-trait-driven | - |
| rs451643 | 8.82E-17 | FGFR4 | Single-trait-driven | Appendicular lean mass |
| rs55666908 | 3.03E-10 | REEP3 | Novel | - |
| rs55747707 | 5.05E-34 | MLXIPL | Known | cholesterol levels, Total fatty acid levels, Serum albumin levels… |
| rs583484 | 8.81E-14 | snoU13 | Single-trait-driven | - |
| rs601338 | 1.63E-33 | FUT2 | Single-trait-driven | CCL25 levels, C-reactive protein levels, Blood metabolite levels… |
| rs62130338 | 7.17E-11 | NTN5 | LD-tagged | - |
| rs6601599 | 1.50E-21 | BLK | Single-trait-driven | - |
| rs7012814 | 5.63E-21 | MIR597 | Known | lipid, Fibrinogen levels, Estimated glomerular filtration rate… |
| rs73942938 | 4.05E-14 | Metazoa_SRP | Single-trait-driven | - |
| rs889398 | 7.70E-13 | NFAT5 | LD-tagged | Lifetime smoking index, Smoking status |

^a^The related phenotypes are those other than BMI, WHR, WHR_adj_BMI, or GSD.

**Supporting Table S11.** Functional annotation for the pleiotropic loci from cross-trait meta-analysis through HeploReg V4.1.

| **SNP** | **Promoter histone marks^1^** | **Enhancer histone marks^2^** | **DNAse^3^** | **Proteins bound^4^** | **Motifs changed^5^** | **dbSNP func annot** |
| --- | --- | --- | --- | --- | --- | --- |
| **BMI and GSD** | |  |  |  |  |  |
| rs10424365 | - | - | - | - | PPAR | intronic |
| rs10882884 | - | - | - | - |  | intronic |
| rs11065363 | - | - | - | - | 9 altered motifs |  |
| rs12900395 | - | 7 tissues | - | - | 9 altered motifs | intronic |
| rs1374915 | - | IPSC, THYM | - | - | Cdx2, HNF4, Nr2e3 |  |
| rs147233090 | - | BLD | - | - | 12 altered motifs |  |
| rs17145750 | - | 4 tissues | - | - | PPAR | intronic |
| rs2798297 | - | BLD | - | - | 8 altered motifs |  |
| rs3744405 | SKIN | 6 tissues | IPSC, LIV | - | 6 altered motifs | intronic |
| rs451643 |  | ESC, GI, MUS | MUS | - |  |  |
| rs4886838 | BLD | 5 tissues | 5 tissues | - | 8 altered motifs | intronic |
| rs6544597 | - | 9 tissues | - | GATA2, P300 |  | intronic |
| rs6744393 | - | 7 tissues | 4 tissues | - | 12 altered motifs |  |
| rs76637437 | - | - | - | - | Pax-4 | intronic |
| rs836509 | - | - | - | - | CACD, Klf4 | intronic |
| rs9571577 | - | - | - | - | Evi-1, Mef2, PLZF | intronic |
| rs9625962 | - | LIV | - | - | HNF1, LUN-1, NF-Y | intronic |
| rs10136491 | - | - | - | - | 6 altered motifs | intronic |
| rs10761785 | - | - | - | - | 16 altered motifs | intronic |
| rs11012737 | - | BRN | - | - | Foxf1, Foxi1 | intronic |
| rs11672660 | 10 tissues | 11 tissues | 18 tissues | ELF1, POL2, ZEB1 | - | intronic |
| rs1260326 | - | LIV | - | - | NRSF | missense |
| rs2954021 | - | 9 tissues | - | - | 4 altered motifs |  |
| rs6001872 | - | BLD, GI, MUS | - | - | - | intronic |
| rs715 | - | - | - | - | RXRA | 3'-UTR |
| rs7412 | SKIN | 4 tissues | - | - | NRSF, Pax-5 | missense |
| rs7550711 | ESDR, BRN, ADRL | 5 tissues | - | - | - | intronic |
| rs786420 | - | - | - | - | 5 altered motifs | intronic |
| rs1048365 | - | - | 6 tissues | - | 7 altered motifs | 3'-UTR |
| rs10858884 | - | - | IPSC | - | Ets | intronic |
| rs11704728 | - | FAT, BLD, GI | - | - | 4 altered motifs | intronic |
| rs12770588 | - | - | - | - | 4 altered motifs | - |
| rs12972158 | - | - | BLD | - | NRSF | - |
| rs17716013 | - | 6 tissues | ESDR,HRT | - | GR | - |
| rs2457445 | - | - | - | - | 5 altered motifs | - |
| rs2639998 | - | GI | - | - | RAR, RXRA | - |
| rs34811474 | - | - | - | - | 6 altered motifs | missense |
| rs362307 | - | 8 tissues | 6 tissues | FOXA1 | ERalpha-a, Nr2f2 | 3'-UTR |
| rs3764002 | - | GI | - | - | BCL | missense |
| rs6486122 | BRST, LIV, LNG | 15 tissues | 5 tissues | GR | 6 altered motifs | intronic |
| rs6744550 | - | THYM | - | - | ERalpha-a, Pou2f2, Sin3Ak-20 | - |
| rs7200764 | BLD | 9 tissues | SKIN |  | ZBTB33, ZBTB7A | - |
| rs889398 | ESC, ESDR, BLD | 4 tissues | 6 tissues | 5 bound proteins | 4 altered motifs | - |
| rs970635 | - | - | - | - | Foxj2, STAT |  |
| **WHR and GSD** | |  |  |  |  |  |
| rs228757 | 7 tissues | 17 tissues | 11 tissues | JUND, SP1, ZNF263 | 4 altered motifs | missense |
| rs2457445 | - | - | - | - | 5 altered motifs | - |
| rs2686189 | SKIN | 13 tissues | 6 tissues | - | Ets | - |
| rs6889220 | - | - | - | - | Foxj2 | intronic |
| rs10761785 | - | - | - | - | 16 altered motifs | intronic |
| rs11012737 | - | BRN | - | - | Foxf1, Foxi1 | intronic |
| rs13429408 | - | 6 tissues | SKIN | - |  | intronic |
| rs1800437 | 12 tissues | 15 tissues | 31 tissues | CTCF, CMYC | 8 altered motifs | missense |
| rs55747707 | 12 tissues | 17 tissues | 25 tissues | 20 bound proteins | HNF4, Mtf1 | intronic |
| rs7550711 | ESDR, BRN, ADRL | 5 tissues |  | - | - | intronic |
| rs10822180 | - |  |  | - | 4 altered motifs | intronic |
| rs10995543 | - |  | BLD | - | Nkx6-1, p300 | - |
| rs1558902 | LNG | 16 tissues | GI | - | GATA | intronic |
| rs2980856 | CRVX | 7 tissues | 6 tissues | 5 bound proteins | CACD, Pax-8, RXRA | - |
| rs3121419 | - |  | - | - | Brachyury | intronic |
| rs36120341 | - | 7 tissues | - | - | 25 altered motifs | intronic |
| rs3764002 | - | GI | - | - | BCL | missense |
| rs429358 | LIV, BRN | IPSC, ESC, ADRL | LIV | - | 10 altered motifs | missense |
| rs6486122 | BRST, LIV, LNG | 15 tissues | 5 tissues | GR | 6 altered motifs | intronic |
| rs702484 | - | 9 tissues | SKIN | - | DMRT2, GR,Sox | intronic |
| rs7193778 | LNG, CRVX, BLD | 17 tissues | 18 tissues | 4 bound proteins | 4 altered motifs | - |
| rs733381 | - | 9 tissues | KID,OVRY | ZNF263 | BCL, p300 | intronic |
| rs73942938 | - | ESDR, LIV | - | - | 4 altered motifs | - |
| rs889398 | ESC, ESDR, BLD | 4 tissues | 6 tissues | 5 bound proteins | 4 altered motifs | - |
| **WHRadjBMI and GSD** | |  |  |  |  |  |
| rs55666908 | - | ESC, IPSC, SKIN | - | - | Egr-1, THAP1, p53 | - |
| rs62130338 | - | - | - | - | 13 altered motifs | intronic |
| rs889398 | ESC, ESDR, BLD | 4 tissues | 6 tissues | 5 bound proteins | 4 altered motifs | - |
| rs112875651 | - | BLD | - | - | 4 altered motifs | - |
| rs13280055 | - | - | - | - | Myc | - |
| rs1883016 | - | BLD, FAT, SPLN | - | - | HNF4, SMC3 | intronic |
| rs3784924 | BLD | 9 tissues | 7 tissues | STAT3 | GR | intronic |
| rs55747707 | 12 tissues | 17 tissues | 25 tissues | 20 bound proteins | HNF4, Mtf1 | intronic |
| rs7012814 | - | LIV | - | - | NRSF, SETDB1, Zfx | - |
| rs1047891 | - | - | - | - | Rad21, SREBP, VDR | missense |
| rs13429408 | - | 6 tissues | SKIN | - | - | intronic |
| rs2256249 | - | GI, HRT | HRT | - | 24 altered motifs | - |
| rs3121419 | - | - | - | - | Brachyury | intronic |
| rs36120341 | - | 7 tissues | - | - | 25 altered motifs | intronic |
| rs3744405 | SKIN | 6 tissues | IPSC, LIV | - | 6 altered motifs | intronic |
| rs3764002 | - | GI | - | - | BCL | missense |
| rs3828944 | 24 tissues | - | 37 tissues | 5 bound proteins | 11 altered motifs | intronic |
| rs429358 | LIV, BRN | IPSC, ESC, ADRL | LIV | - | 10 altered motifs | missense |
| rs4454603 | - | 10 tissues | SKIN | - | - | intronic |
| rs451643 | - | ESC, GI, MUS | MUS | - | - | - |
| rs583484 | - | - | - | - | - | - |
| rs601338 | - | - | - | - | HDAC2, HNF4 | nonsense |
| rs6601599 | - | BLD | BLD | - | 6 altered motifs | intronic |
| rs73942938 | - | ESDR, LIV | - | - | 4 altered motifs | - |

^1^Evidence of local H3K4Me1 and H3K27Ac modification (cell lines/types: if >3, only the number is included).

^2^Evidence of local H3K4Me3 modification (cell lines/types: if >3, only the number is included).

^3^Evidence of chromatin hypersensitivity to DNase (cell lines/types: if >3, only the number is included).

^4^ChIP-seq experiments indicate alteration in binding of transcription factor ( if >3, only the number is included).

^5^Evidence of alteration in regulatory motif ( if >3, only the number is included).

Section B: **Supporting** Figures


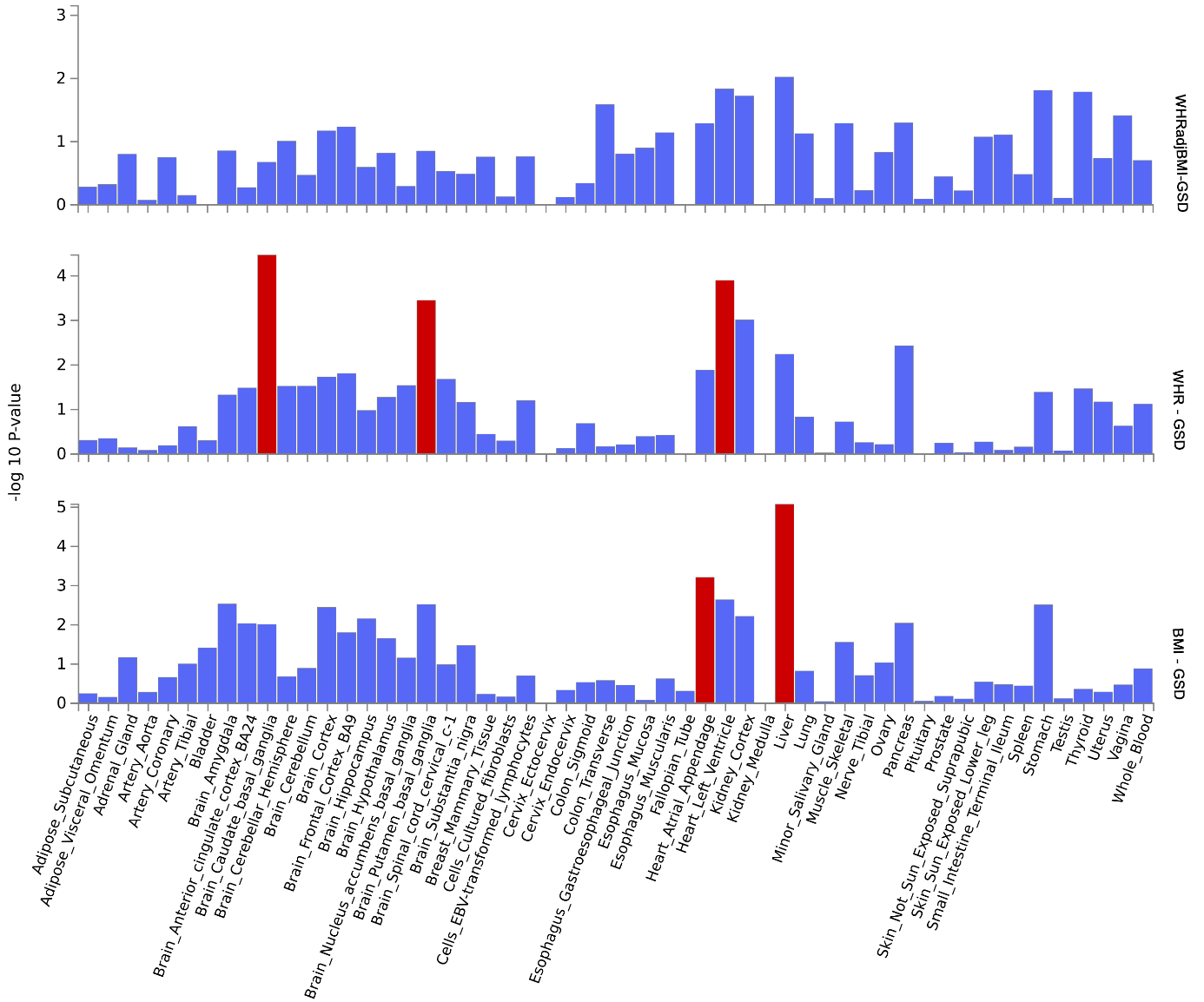


**Figure S1.** GTEx tissue enrichment analysis for expression of the shared genes underlying obesity and gallstone disease. Red represents significant tissue enrichment after Benjamin-Hochberg correction. BMI, body mass index; GSD, gallstone disease; WHR, waist-to-hip ratio; WHR_adj_BMI, waist-to-hip ratio adjusted for body mass index.


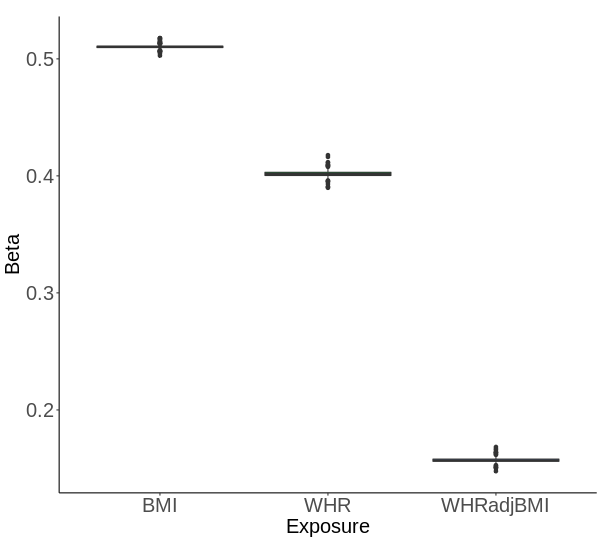


**Figure S2.** Box plot of betas in leave-one-out analysis in the Mendelian randomization. Beta: effect allele beta coefficient. BMI, body mass index; WHR, waist-to-hip ratio; WHR_adj_BMI, waist-to-hip ratio adjusted for body mass index.


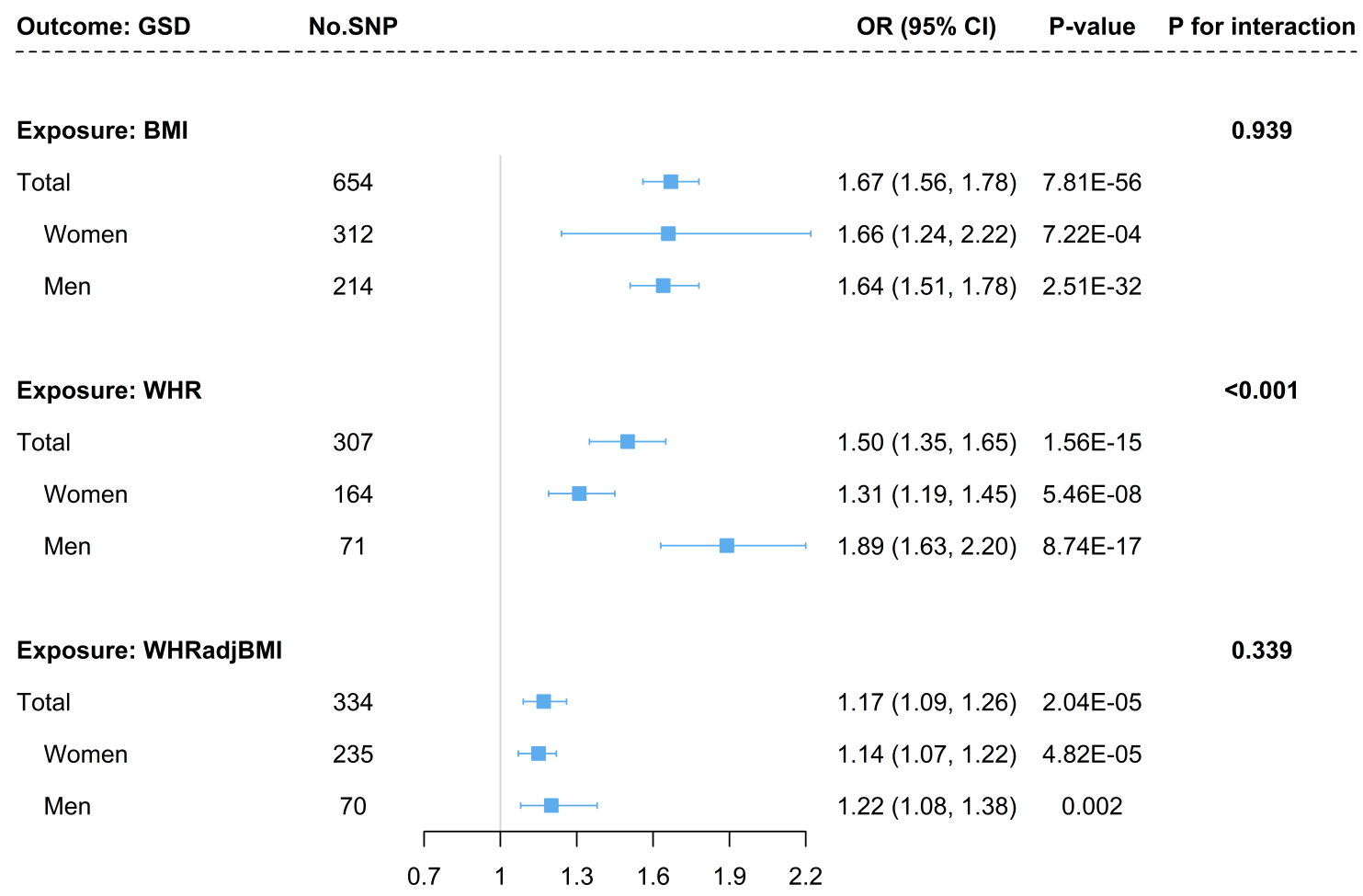


**Figure S3.** Estimates of causal effect sizes for sex-specific Mendelian randomization. GSD, gallstone disease; BMI, body mass index; WHR, waist-to-hip ratio; WHR_adj_BMI, waist-to-hip ratio adjusted for body mass index





**Figure S4.** Estimates of causal effect sizes for genetical predisposition to gallstone disease on obesity-related traits. GSD, gallstone disease; BMI, body mass index; WHR, waist-to-hip ratio; WHR_adj_BMI, waist-to-hip ratio adjusted for body mass index.

**Reference**

1. Li R, Li R, Xie J, et al. Associations of socioeconomic status and healthy lifestyle with incident early-onset and late-onset dementia: a prospective cohort study. Lancet Healthy Longev 2023;4(12):e693-e702.

2. Madjedi KM, Stuart KV, Chua SYL, et al. The Association of Physical Activity with Glaucoma and Related Traits in the UK Biobank. Ophthalmology 2023;130(10):1024-1036.

3. Choi J, Wen W, Jia G, et al. Lifestyle factors, genetic susceptibility to obesity and their interactions on coronary artery disease risk: A cohort study in the UK Biobank. Prev Med 2024;180:107886.

4. Xiang Y, Xu H, Chen H, et al. Tea consumption and attenuation of biological aging: a longitudinal analysis from two cohort studies. Lancet Reg Health West Pac 2024;42:100955.

5. Lv X, Cai J, Li X, et al. Body composition, lifestyle, and depression: a prospective study in the UK biobank. BMC Public Health 2024;24(1):393.

6. Celis-Morales CA, Lyall DM, Gray SR, et al. Dietary fat and total energy intake modifies the association of genetic profile risk score on obesity: evidence from 48 170 UK Biobank participants. Int J Obes (Lond) 2017;41(12):1761-1768.

7. Cuevas A, Miquel JF, Reyes MS, et al. Diet as a risk factor for cholesterol gallstone disease. J Am Coll Nutr 2004;23(3):187-196.

8. Skrivankova VW, Richmond RC, Woolf BAR, et al. Strengthening the Reporting of Observational Studies in Epidemiology Using Mendelian Randomization: The STROBE-MR Statement. JAMA 2021;326(16):1614-1621.

9. Skrivankova VW, Richmond RC, Woolf BAR, et al. Strengthening the reporting of observational studies in epidemiology using mendelian randomisation (STROBE-MR): explanation and elaboration. BMJ 2021;375:n2233.
